# Finite-Size Effects in Nonlocal Metasurfaces

**DOI:** 10.1021/acsphotonics.6c00860

**Published:** 2026-09-02

**Authors:** Tom Hoekstra, Sander A. Mann, Jorik van de Groep

**Affiliations:** Van der Waals-Zeeman Institute, Institute of Physics, 1234 University of Amsterdam, Amsterdam 1098 XH, The Netherlands

**Keywords:** nonlocal metasurfaces, guided-mode resonances, finite-size effects, spatiotemporal coupled-mode theory, 2D materials

## Abstract

Metasurfaces leveraging nonlocal resonances enable narrowband spectral control and strong near-fields, with applications spanning augmented reality, biosensing, and nonlinear optics. However, the large spatial extent of these modes also poses new challenges: finite-size effects often deteriorate the performance of practical, footprint-limited devices. Here, we develop a spatiotemporal coupled-mode theory model that intuitively and quantitatively captures how finite size affects the scattering response of nonlocal metasurfaces. This reveals that, when the modal propagation length becomes constrained by the physical interaction length, the scattered field shows strong interference fringes and line width broadening. We derive an expression for the quality factor that incorporates an additional edge-loss channel, demonstrating that the stored energy and effective lifetime scale exponentially with the interaction length. We validate these predictions experimentally using position- and momentum-resolved spectroscopy on a 30-μm-wide metasurface. Overall, this work formalizes the impact of finite size on the scattering response of nonlocal photonic systems, and provides handles on how to minimize the impact of finite-size effects in metasurface design.

## Graphical abstract

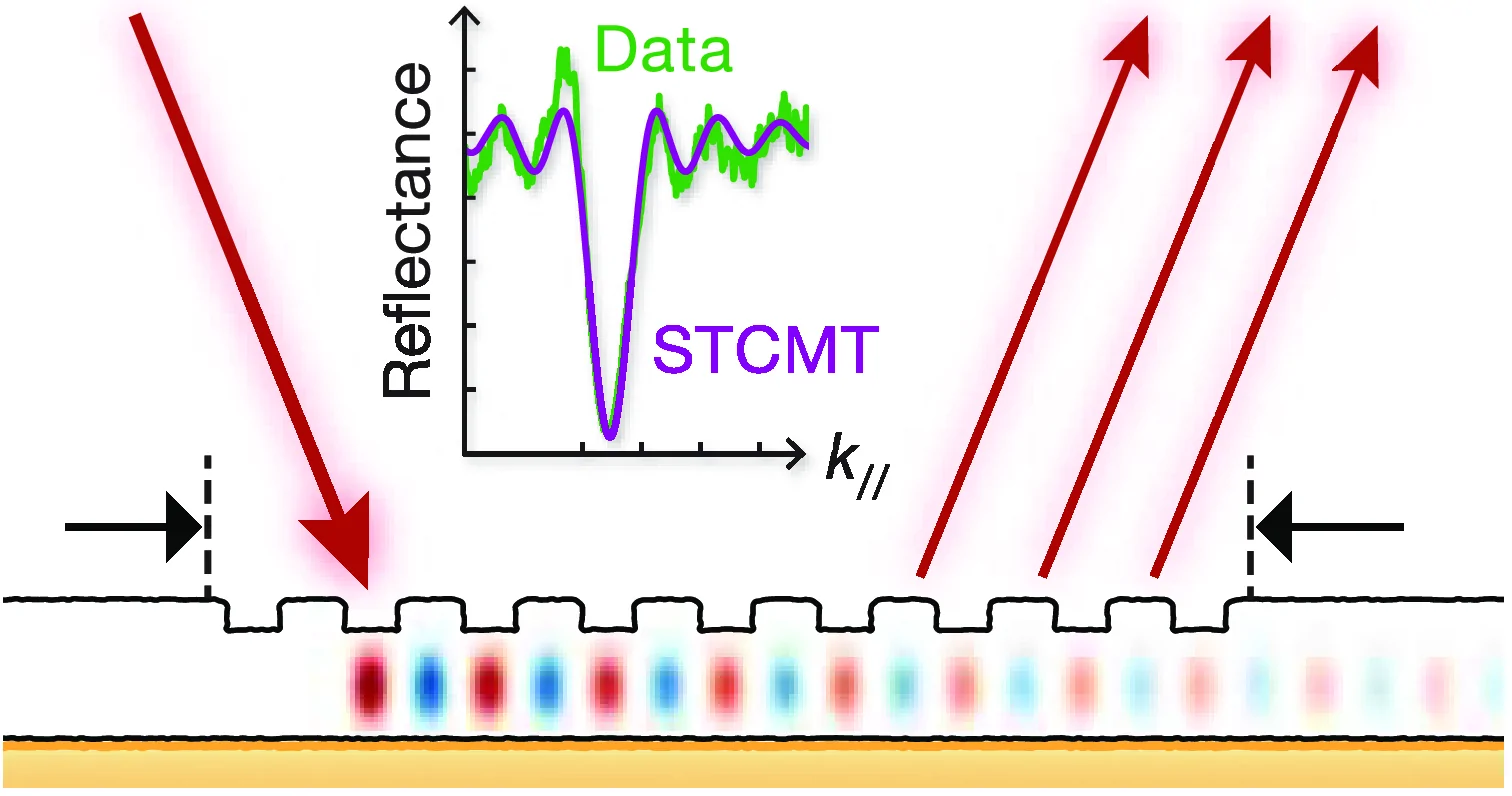

## Introduction

Over the past decades, planar arrays of resonant nanostructures—metasurfaces—have evolved from simple optical components mimicking their bulk counterparts to complex systems performing a multitude of novel functions.^1,2^ Using a variety of materials and geometries to tailor the scattering response of dielectric or metallic nanostructures, local metasurfaces can sculpt the spatial phase profile of an impinging wavefront with deeply subwavelength resolution. Increasingly, though, optoelectronic technologies require narrowband selectivity and strong light–matter interactions that are difficult to achieve with deeply subwavelength meta-atoms, whose quality (*Q*) factors tend to be low.

Nonlocal metasurfaces, which harness spatially extended modes such as guided-mode resonances^3–5^ and quasi-bound states in the continuum,^6–8^ have emerged as a promising alternative to local metasurfaces.^9^ These long-lived resonant states can support extremely high *Q*-factors,^10–12^ thereby delivering tremendous spectral resolution and near-field enhancements. Recently, it was even shown that nonlocalities can be engineered with locally varying features to simultaneously command both the spectral and spatial degrees of freedom.^13–15^ Combined, these properties enable wide-ranging applications in biomolecular sensing,^16–18^ coherent light sources,^19–21^ free-space modulators,^22–24^ mixed-reality eyewear,^25,26^ nonlinear optics,^27–29^ optical image processing,^30,31^ thermal metasurfaces,^32,33^ and wavefront shaping.^34–36^ It is therefore compelling to embrace nonlocal metasurfaces in the move toward ultracompact optical devices.

In the pursuit of miniaturizing meta-optical devices, the lateral dimensions of metasurfaces are increasingly compressed to fit within limited footprints. Whereas the impact of finite sizes can largely be ignored for local metasurfaces, with only select works investigating them,^37–40^ the inherent delocalization of high-*Q* resonances in nonlocal metasurfaces implies that finite-size effects can no longer be neglected.^28,41,42^ Indeed, not long after the introduction of guided-mode resonant gratings,^43,44^ it was realized that finite sizes can degrade their performance.^45–53^ While these early works established the importance of finite-size effects on the resonance line width, they were often limited to empirical observations, system-specific approximations, or limited to simple scaling laws for the effective modal propagation length,^52^ lacking a unified modeling framework that quantifies the spatially dependent excitation as well as the spectral fringes that are commonly observed around the resonance frequency. This trend persists today, as nonlocal metasurfaces are still typically simulated as infinitely periodic because finite arrays come with excessive computational costs in full-wave solvers and are not naturally captured using temporal coupled-mode theory.^54,55^ As a result, the resonant response of fabricated devices is often influenced by the unpredictable corollaries of finite size, which has already motivated mitigation strategies^41,56–58^ and theoretical investigations.^59,60^ Nevertheless, a broadly applicable predictive modeling framework has so far remained elusive.

Here, we develop a model that intuitively and quantitatively captures how a finite lateral footprint reshapes the scattering response of guided-mode resonant metasurfaces. We use spatiotemporal coupled-mode theory (STCMT), a recent extension of temporal coupled-mode theory that captures spatial inhomogeneity.^61–63^ Our model explicitly accounts for excitation of a traveling wave that can reradiate within a finite aperture due to the limited grating width. We show that when the physical interaction length becomes comparable to the modal propagation length, finite-size effects fundamentally modify the optical response, giving rise to excitation position-dependent interference fringes and line width broadening. Crucially, we derive expressions for the effective *Q*-factor and stored energy, demonstrating that both scale exponentially with the interaction length *L*, which is defined as the distance over which the mode interacts with the grating after excitation at *x*_0_, i.e., the scattering aperture (Figure 1a). We validate the model experimentally by fabricating a 30 μm-wide metasurface and performing position- and momentum-resolved reflectance spectroscopy. The measurements show remarkable agreement with the theory, directly confirming the predicted size effects and enabling quantitative extraction of the intrinsic and radiative loss rates while accounting for the finite scattering aperture. Altogether, this work establishes STCMT as an invaluable tool for designing and interpreting footprint-limited nonlocal metasurfaces.

**Figure 1:**
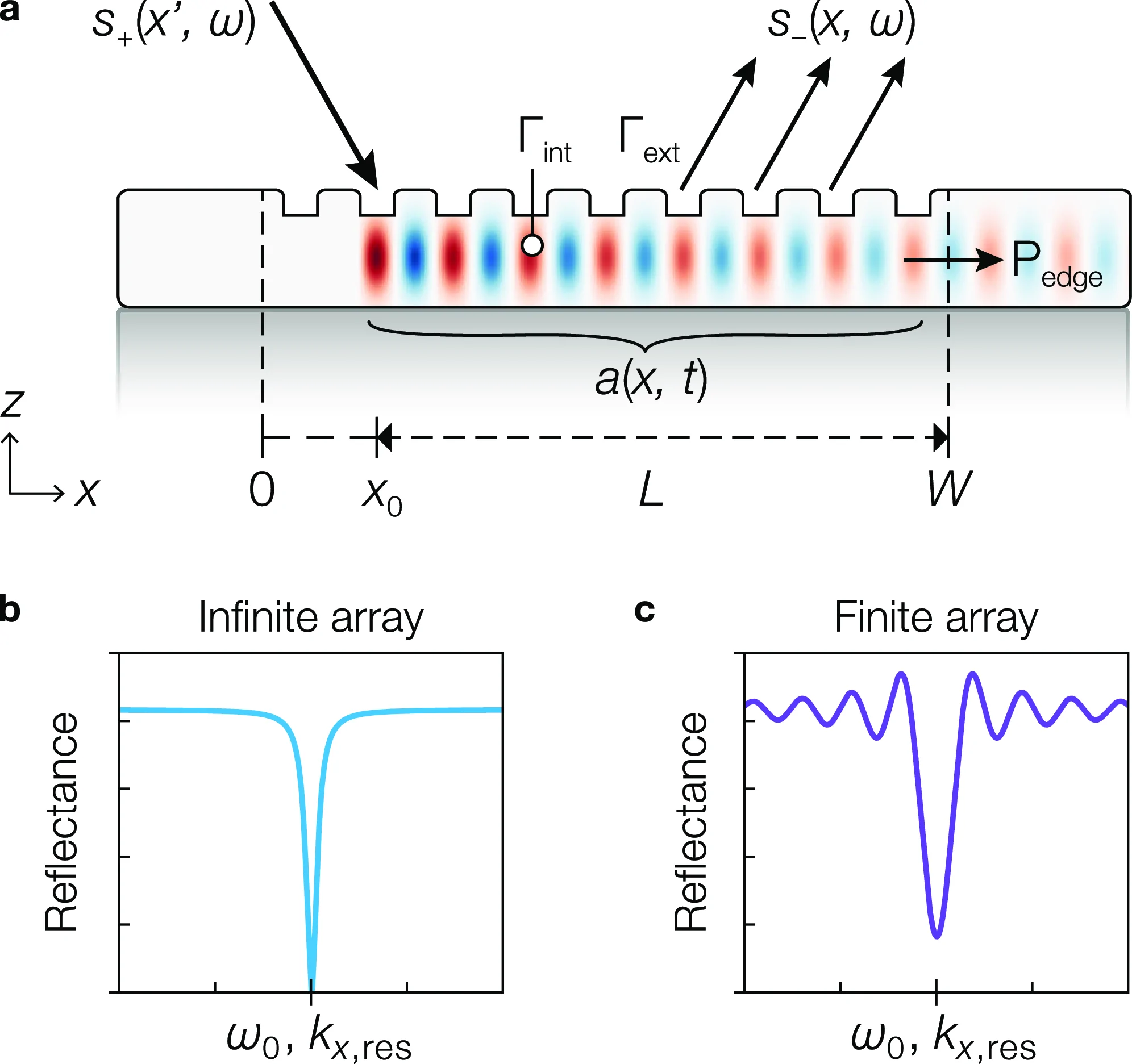
Nonlocal metasurface with finite-size effects. (a) Incident field *s*_+_(*x*′, ω) centered at *x*_0_ excites a right-propagating quasi-guided mode with amplitude *a*(*x*, *t*). The mode has an interaction length *L*, over which it decays through intrinsic loss (Γ_int_) and reradiation (Γ_ext_). Power reaching the right edge of the grating at *x* = *W* cannot radiate to free-space, and is represented by power flux *P*_edge_. The scattered field is denoted *s*_–_(*x*, ω). (b) Modeled reflectance for an infinite, and (c) a finite metasurface array, respectively, showing the impact of finite size on the scattering response near the resonant frequency ω_0_ or in-plane wavevector *k*_*x*,res_.

## Results

In this work, we study the effects of finite size on the optical response of a reflective guided-mode resonator. Here, the structure consists of a waveguide backed by a reflector and with a subwavelength grating of period Λ patterned over a finite width *W* (Figure 1a). The scattering problem is described in terms of a single modal parameter *a*(*x*, *t*) that is excited from free-space by a spatially localized input field *s*_+_(*x*′). Compared to an infinite metasurface, lattice truncation can fundamentally alter the scattered field *s*_–_(*x*) near the resonant frequency ω_0_ or, equivalently, the in-plane wavevector *k*_*x*,res_, introducing fringes and broadening the resonance (Figure 1b,c).

To determine the origin of the finite-size effects observed in Figure 1c, we first start with an infinitely periodic system. We consider a quasi-guided mode that is characterized by a dispersion that is linearized around a point (ω_0_, β_0_) as

1 $$\Omega \left(\beta \right)≃\omega_{0}-i\Gamma +v_{g}\left(\beta -\beta_{0}\right)$$

where β is the phase constant, *v*_g_ is the group velocity, and Γ = Γ_int_ + Γ_ext_ is the rate at which the mode decays due to internal (nonradiative) and external (radiative) mechanisms, respectively (Figure 1a). To achieve such dispersion, we design a dielectric hexagonal boron nitride (hBN) waveguide on a gold (Au) back-reflector that supports the fundamental TE_0_ mode (Figure 2a). The waveguide is capped with a low-index dielectric polymer, specifically chemically semiamplified resist (CSAR), in which a subwavelength grating is patterned with period Λ = 368 nm and duty cycle of 36%. In this system, the duty cycle governs the radiative coupling (Γ_ext_), while nonradiative losses (Γ_int_) are incurred evanescently in the Au layer. We optimize the geometry to achieve mirror-like background reflection over the relevant bandwidth.

**Figure 2:**
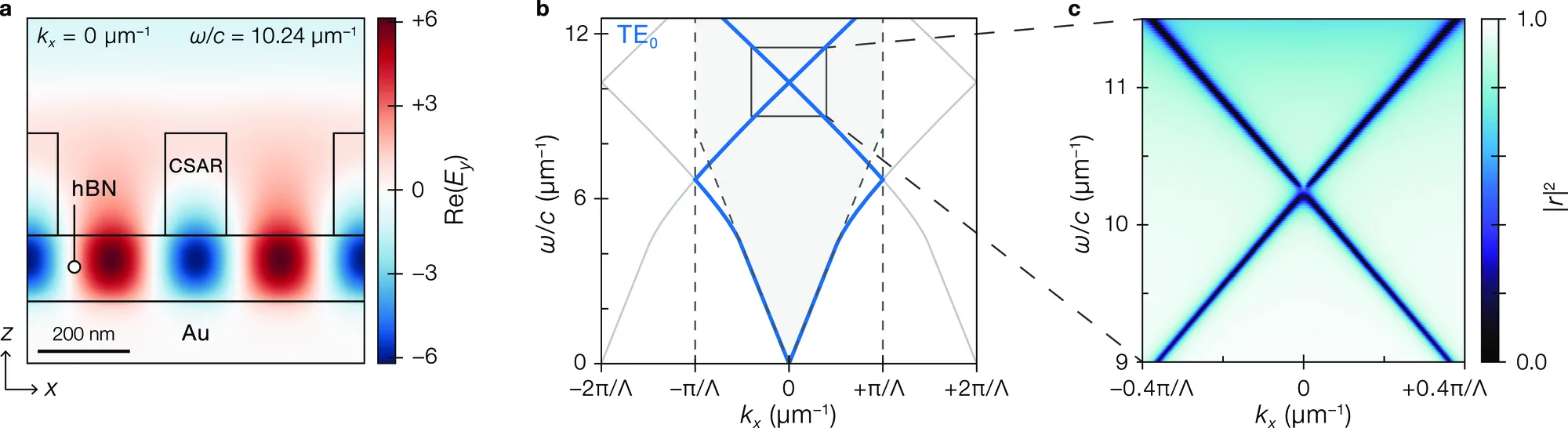
Semi-infinite guided-mode resonator. (a) Full-wave simulation of the electric-field profile Re­(*E*_*y*_) of the quasi-guided mode in a resonator comprised of a semi-infinite CSAR subwavelength grating on a hBN waveguide with an Au back-reflector. (b) Calculated TE_0_ guided-mode dispersion, where the grating is modeled as an effective medium. The grating folds the dispersion into the first Brillouin zone (−π/Λ to +π/Λ), shown by vertical dashed lines, with the portion that lies within the light cone indicated by the gray shaded region. (c) Simulated momentum-resolved reflectance (|*r*|^2^) showing the corresponding dispersion.

The periodic perturbation in the CSAR layer breaks the in-plane translational symmetry and folds the dispersion of the guided mode into the light cone (Figure 2b). The subwavelength periodicity restricts scattering to the zeroth-order direct reflection channel, with the diffracted orders constituting both left- and right-propagating evanescent waves owing to the grating’s symmetric design. From full-wave simulations (Figure 2c), we find that the coupling between these modes is negligible except in the vicinity of *k*_*x*_ = 0 μm^–1^, and consequently, we can treat them as independent linear modes over most of the relevant *k*_*x*_ range (this is verified in Supporting Information Figure S2). This is not a general result, and a parabolic dispersion arises at the band edge when the coupling between forward and backward propagating modes is non-negligible.^61^ We attribute the minimal mode coupling to their predominant confinement in the continuous waveguide layer (Figure 2a) and to the relatively weak scattering efficiency of the low-contrast grating.

The dispersion is obtained numerically using an eigenmode solver by modeling the grating as an effective medium, which directly yields most of the physical parameters in the dispersion relation, eq 1. However, since the eigenmode is computed for a closed system, we must obtain Γ_ext_ by fitting the simulated response of the driven structure (Figure 2c). While Γ_ext_ may in principle depend on frequency or be modified by mode hybridization, we find that a constant value of Γ_ext_ = 7.4 × 10^12^ s^–1^ is sufficient to accurately reproduce the full-wave response over the bandwidth considered here (Supporting Information Figure S2).

The decay rate sets the resonance lifetime and is closely related to the quality factor, which is formally defined as the ratio of stored energy *U* in the resonator to the rate of energy dissipation *P*_loss_,

2 $$Q\equiv \omega_{0}\frac{U}{P_{\mathrm{loss}}}=\frac{\omega_{0}}{2\Gamma }$$

as well as the modal propagation length,

3 $$L_{p}=\frac{v_{g}}{\Gamma }$$

which governs the characteristic length scale over which nonlocality must be considered (i.e., the nonlocality length^62^). When an impinging wave launches a right-propagating mode at *x*′ = *x*_0_, the resulting excitation interacts with the structure over a finite length *L* = *W*–*x*_0_. In the semi-infinite limit, *L*/*L*_p_ → ∞, the array size negligibly perturbs the resonant response (Figure 1b). Conversely, in the short-grating limit, *L* ≲ *L*_p_, finite-size effects critically impair the theoretical performance (Figure 1c). At ω/*c* = 9 μm^–1^, we find *L*_p_ = 11.9 μm and *Q* = 132, which reduces to *L*_p_ = 6.4 μm and *Q* = 96 at ω/*c* = 11.5 μm^–1^ due to increased optical losses in the Au layer at higher frequencies.^64^

The infinitely periodic limit discussed so far is typically the starting point of metasurface design, but it is clear that if the lateral dimensions of the metasurface are close to the propagation length *L*_*p*_, then, we may expect significant finite-size effects. In the following, we will discuss how to take these finite-size effects into consideration using STCMT.

### Finite-Size Effects

Generally, the spatiotemporal equation of motion for a system with one input port and a locally linear dispersion is written as^62^

4 $$\begin{array}{l} \frac{\partial a\left(x,t\right)}{\partial t}+v_{g}\frac{\partial a\left(x,t\right)}{\partial x}+i\left(\omega_{0}-i\Gamma -v_{g}\beta_{0}\right)a\left(x,t\right) \end{array}=\int \kappa \left(x,x^{\prime }\right)s_{+}\left(x^{\prime },t\right)dx^{\prime }$$

This equation describes the propagation of a single modal amplitude *a*(*x*, *t*) that is excited from free-space by an incident wave *s*_+_(*x*′, *t*) with coupling coefficient κ. The parameter *a*(*x*, *t*) is normalized such that its absolute value squared gives the instantaneous stored energy in the nonlocal mode per unit area, while |*s*_+_|^2^ captures the input power density. As the mode propagates along the structure, it radiates to free-space (with coupling described by *d*) to build up the scattered field *s*_–_(*x*, *t*) (also normalized such that |*s*_–_|^2^ is the outgoing power density) in interference with the nonresonant background, described by *Cs*_+_(*x*′, *t*) (additional details in Supporting Information Section S2):

5 $$s_{-}\left(x\right)=\int \left(C\left(x,x^{\prime }\right)s_{+}\left(x^{\prime }\right)+d\left(x,x^{\prime }\right)a\left(x^{\prime }\right)\right)dx^{\prime }$$

Although the mode has a nonlocal spatial distribution, we assume scattering is purely localized.^62^ As a result, the scattering coefficients *C*(*x*, *x*′), κ­(*x*, *x*′), and *d*(*x*, *x*′) become proportional to δ­(*x* – *x*′) (Supporting Information Section S2). In the steady state, eqs 4 and 5 then reduce to

6 $$v_{g}\left(\frac{\partial }{\partial x}-i\mathrm{\beta ̃}\right)a\left(x\right)=\kappa \left(x\right)s_{+}\left(x\right)$$

7 $$s_{-}\left(x\right)=-s_{+}\left(x\right)+d\left(x\right)a\left(x\right)$$

where we introduced the complex propagation constant β̃ = β + *iα* with attenuation rate α = 1/*L*_p_ = Γ/*v*_g_ in the standard waveguide form. In addition, we assumed a perfectly reflective off-resonant response over the bandwidth of interest, i.e., *C* = −1.

To apply the STCMT framework to finite-size effects that arise upon truncating the metasurface grating to a width of *W*, we simply take the coupling coefficients to be nonzero in space only over the same width,

8 $$\kappa \left(x\right)=d{\left(x\right)}^{*}=\sqrt{2\Gamma_{\mathrm{ext}}}e^{\mathrm{iqx}}\Theta \left(x\right)\Theta \left(W-x\right)$$

where $\sqrt{2\Gamma_{\mathrm{ext}}}$ is the familiar radiative coupling term from temporal coupled-mode theory^54^ and the phase factor (*e* ^*iqx*^, with *q* = 2π/Λ) encodes in-plane momentum matching between the guided mode and free-space radiation. Crucially, the Heaviside functions (Θ) restrict coupling to the finite interval *x* ∈ [0, *W*]. Note that the standard TCMT identities |κ|^2^ = |*d*|^2^ = 2Γ_ext_ and Cd* = −κ are satisfied. For the reference-plane convention used here (*C* = −1), this relation reduces to κ = d*. This is consistent with the fact that in-coupling from the incident wave to the guided mode supplies the grating momentum *q*, whereas out-coupling from the guided mode to the reflected wave removes *q*. The two coefficients therefore carry opposite spatial phase factors. This is different from the conventional reciprocal TCMT relation κ = *d*, which assumes a modal basis invariant under time-reversal. The traveling guided mode considered here carries nonzero momentum and, under time-reversal, is mapped onto the mode with opposite momentum. Microscopic reversibility therefore relates the in-coupling coefficient of the mode at β to the out-coupling coefficient of its time-reversed partner at −β, such that κ_β_ = *d*_–β_ rather than κ_β_ = *d*_β_.^65^

To capture the essential physics of interest here, we find that it is sufficient to model *only* a single, right-propagating mode,

9 $$a\left(x\right)=\frac{\sqrt{2\Gamma_{\mathrm{ext}}}}{v_{g}}\int_{0}^{x}e^{i\mathrm{\beta ̃}\left(x-x\prime \right)}e^{\mathrm{iqx}\prime }s_{+}\left(x^{\prime }\right)dx^{\prime }$$

While this simplification strictly speaking violates reciprocity (due to the absence of a backward propagating mode), we find this approach justified in the regime where the interaction between the forward and backward modes is negligible. In the designed metasurface, owing to the low-index-contrast grating, this condition is satisfied except near *k*_*x*_ = 0 μm^–1^ (Figure 2c), which allows the two modes to be treated as approximately independent linear branches over most of the relevant momentum range. However, the approximation may break down in higher-index-contrast systems, where distributed coupling can substantially modify the dispersion and both counter-propagating modes must be included explicitly.^61^ We emphasize that the STCMT framework remains applicable in this regime, but the scalar Green’s function must be replaced by a matrix-valued formulation describing the coupled modes.

The preceding equations provide us with a method to solve the reflected field from a finite nonlocal metasurface. In general, the easiest way to solve for the reflection involves numerically integrating eq 9 to find the mode amplitude, and then inserting this into eq 5. In certain cases, however, these equations can readily be solved analytically: for example, when the resonance line width is much narrower than the NA of excitation (α ≪ *k*_*x*,max_), we can approximate the diffraction-limited input field as a δ-function (the validity of this approximation is confirmed in Supporting Information Figure S3). This collapses the reflection coefficient *r*(*k*_*x*_) = *S*_–_(*k*_*x*_)/*S*_+_(*k*_*x*_) to a compact closed form,

10 $$r\left(k_{x}\right)=-1+\frac{2\Gamma_{\mathrm{ext}}}{v_{g}\left(\alpha +i\Delta_{k}\right)}\left(1-e^{-\left(\alpha +i\Delta_{k}\right)L}\right)$$

or similarly in terms of ω (full derivation in Supporting Information Section S3). Here, the first factor recovers the familiar Lorentzian dependence on detuning Δ_*k*_ = *k*_*x*_–*k*_*x*,res_, while the exponential term encodes the effects of finite size via the interaction length *L*. The prevalence of these effects is governed by the relative magnitudes of *L* and *L*_p_. In the short-grating limit, *L* ≲ *L*_p_, the finite aperture produces oscillatory spectral features and apparent resonance broadening, whereas in the long-grating limit, *L* ≫ *L*_p_, the finite-size term vanishes and eq 10 reduces to the standard one-port temporal coupled-mode expression.^54^

In the following, we apply the model to a finite metasurface with width *W* = 30 μm illuminated by a coherent, diffraction-limited input field arising from the Fourier transform of a one-dimensional entrance pupil with numerical aperture NA = *k*_*x*,max_/*k*_0_ = 0.4. The excitation launches a right-propagating quasi-guided mode that decays through absorption and radiation. As the mode reaches the edge of the grating at *x* = *W*, any remaining power cannot radiate to the far-field as it continues propagating in the unpatterned region (quantified by *P*_edge_). The finite width *W* thus restricts the mode’s interaction length, or scattering aperture, to *L* = *W*–*x*_0_. However, for metasurfaces with more abrupt and strongly reflective terminations, the reflected wave can interfere coherently with the counter-propagating mode excited directly by the incident beam, making the response dependent on both the amplitude and phase of the edge reflection. If both edges are reflective and the propagation length is long enough for repeated round trips, Fabry-Pérot-like standing-wave resonances in the plane of the metasurfaces could occur. Engineering the phase and amplitude of such edge reflections therefore offers opportunities for high-Q metasurfaces within footprint-limited geometries.^56^

Figure 3a shows the input and output fields at frequency *k*_0_ = ω/*c* = 9.7 μm^–1^ and centered at *x*_0_ = 22 μm, corresponding to an interaction length *L* = 8 μm. We observe strongly localized excitation with characteristic sinc fringes (in salmon), and a reflected field that is only slightly reduced in amplitude (in purple), since most of the incident power is distributed among nonresonant *k*-vectors. However, when looking at the mode amplitude, we clearly observe an excitation that is launched at *x*_0_, right-propagates and exponentially decays (Figure 3a, inset). At this frequency, the propagation length is *L*_p_ = 10.6 μm such that the mode amplitude only reduces to *e* ^–*L*/*L*_p_^ ≈ 0.47 before reaching the edge of the scattering aperture at *x* = *W*, and finite-size effects cannot be ignored.

**Figure 3:**
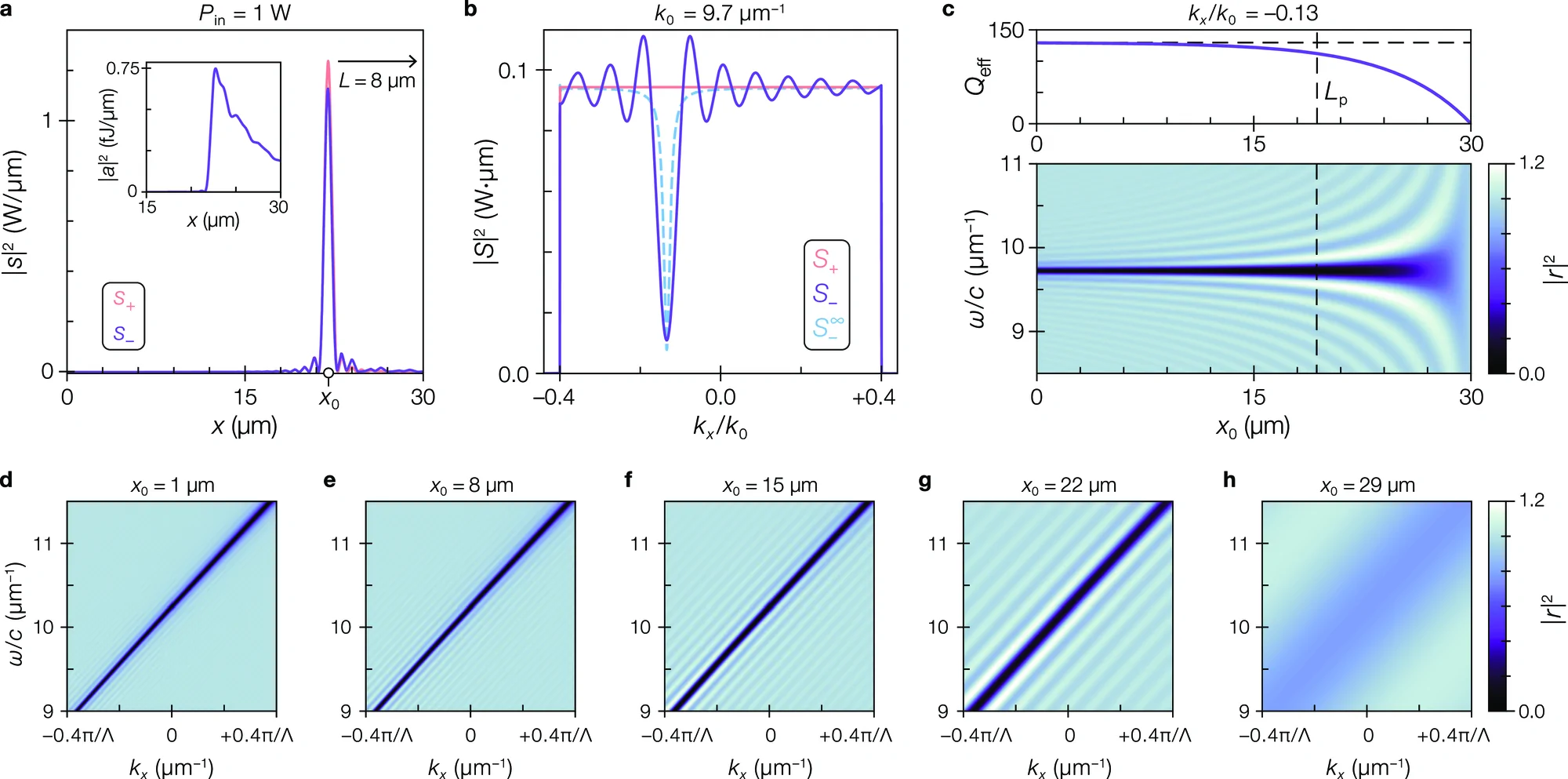
Spatiotemporal coupled-mode theory of a finite-size guided-mode resonator. (a) Real-space input field |*s*_+_(*x*′)|^2^ (salmon) and the corresponding scattered field |*s*_–_(*x*)|^2^ (purple), calculated for a *P*_in_ = 1 W excitation at *x*_0_ = 22 μm (*L* = 8 μm) and NA = *k*_*x*,max_/*k*_0_ = 0.4. The inset shows that the modal amplitude *a*(*x*) is not fully decayed at *x* = *W*. (b) Corresponding transverse-momentum representation of the input field |*S*_+_(*k*_*x*_)|^2^. The resulting scattered field |*S*_–_(*k*_*x*_)|^2^ shows broadening and fringes arising from the finite scattering aperture when compared to the semi-infinite response |*S*_–_^∞^|^2^ (blue, dashed). (c) Evolution of *Q*_eff_ (top) and reflectance (bottom) as a function of *x*_0_, for fixed *k*_*x*_/*k*_0_ = −0.13, with the propagation length *L*_p_ indicated with respect to the edge of the grating (vertical, dashed). *Q*_eff_ tends to the semi-infinite limit (horizontal, dashed) as *L* increases. (d–h) Corresponding reflectance dispersion showing *x*_0_-dependent interference fringes and line widths.

While, in real-space, the effects of lattice truncation appear to be mild, the same cannot be said about Fourier space (Figure 3b). The angular scattering response still exhibits a resonant dip at *k*_*x*_/*k*_0_ = −0.13 (in purple), consistent with the semi-infinite structure (in dashed blue), but the overall response deviates strongly from a simple Lorentzian line shape. Instead, the resonant feature is modulated by a sinc-like envelope set by the finite scattering aperture, with pronounced fringes arising from coherent interference between radiation emitted from different positions along the grating. We note that |*r*(*k*_*x*_)|^2^ can locally exceed unity due to a redistribution of power within the angular spectrum and does not imply optical gain; the total scattered power is limited by the incident power minus the power dissipated through absorption and the power that “escapes” at the edge of the patterned region.

Moving the point of excitation along the sample changes *L* and systematically reshapes the spectral (Figure 3c) and angular response (Figure 3d–h). As the beam approaches the right edge (*x*_0_ → *W*), the interference pattern becomes more pronounced and the fringe spacing increases as Δ*k*_fringe_ = 2π/*L*, in direct analogy with Fraunhofer diffraction from a rectangular aperture. Conversely, moving *x*_0_ farther away from the right edge reduces the fringe spacing. Ultimately, at the left edge (*L* = 29 μm), the response appears indistinguishable from the infinite array (Figure 3d). In this case, the normalized propagation length is *L*/*L*_p_ = 2.7 and the mode decays to *e* ^–*L*/*L*_p_^ ≈ 0.06 before reaching *x* = *W*—providing an indication of the relative sizes required to obtain unperturbed performance.

Due to finite-size broadening, the *Q*-factor cannot be inferred directly from the spectral width of the resonant feature. We therefore define an effective quality factor for the finite scattering aperture from the stored energy and total power leaving this region,

11 $$Q_{\mathrm{eff}}\left(L\right)\equiv \omega_{0}\frac{U}{P_{\mathrm{loss}}+P_{\mathrm{edge}}}=\frac{\omega_{0}}{2\Gamma }\left(1-e^{-2L/L_{p}}\right)$$

where *P*_edge_ = *v*_g_|*a*(*W*)|^2^ is the guided power crossing the boundary of the patterned region. The resulting *Q*_eff_ vanishes as *L* → 0, while approaching the semi-infinite limit (*Q*_eff_ → 130 at *k*_0_ = 9.7 μm^–1^) as *L* ≫ *L*_p_ (Figure 3b). Together, these findings underscore the detrimental impact that finite size can have, even for a resonance with a moderate semi-infinite *Q*-factor.

### Experimental Validation

To fabricate the designed metasurface, we use a dry-transfer technique^66^ to stamp 143 nm-thick mechanically exfoliated hBN onto a prepatterned 30 μm-wide Au back-reflector. We then spin-coat a 222 nm layer of CSAR and pattern the subwavelength grating via electron-beam lithography. Here, we utilize the etch-free approach—where the grating is patterned in a low-index resist rather than shallow-etched in the waveguide itself—to minimize fabrication imperfections and allow rapid prototyping.^11,12,24,67^ Atomic force microscopy reveals excellent uniformity and confirms that the fabricated grating matches the design (Figure 4a).

**Figure 4:**
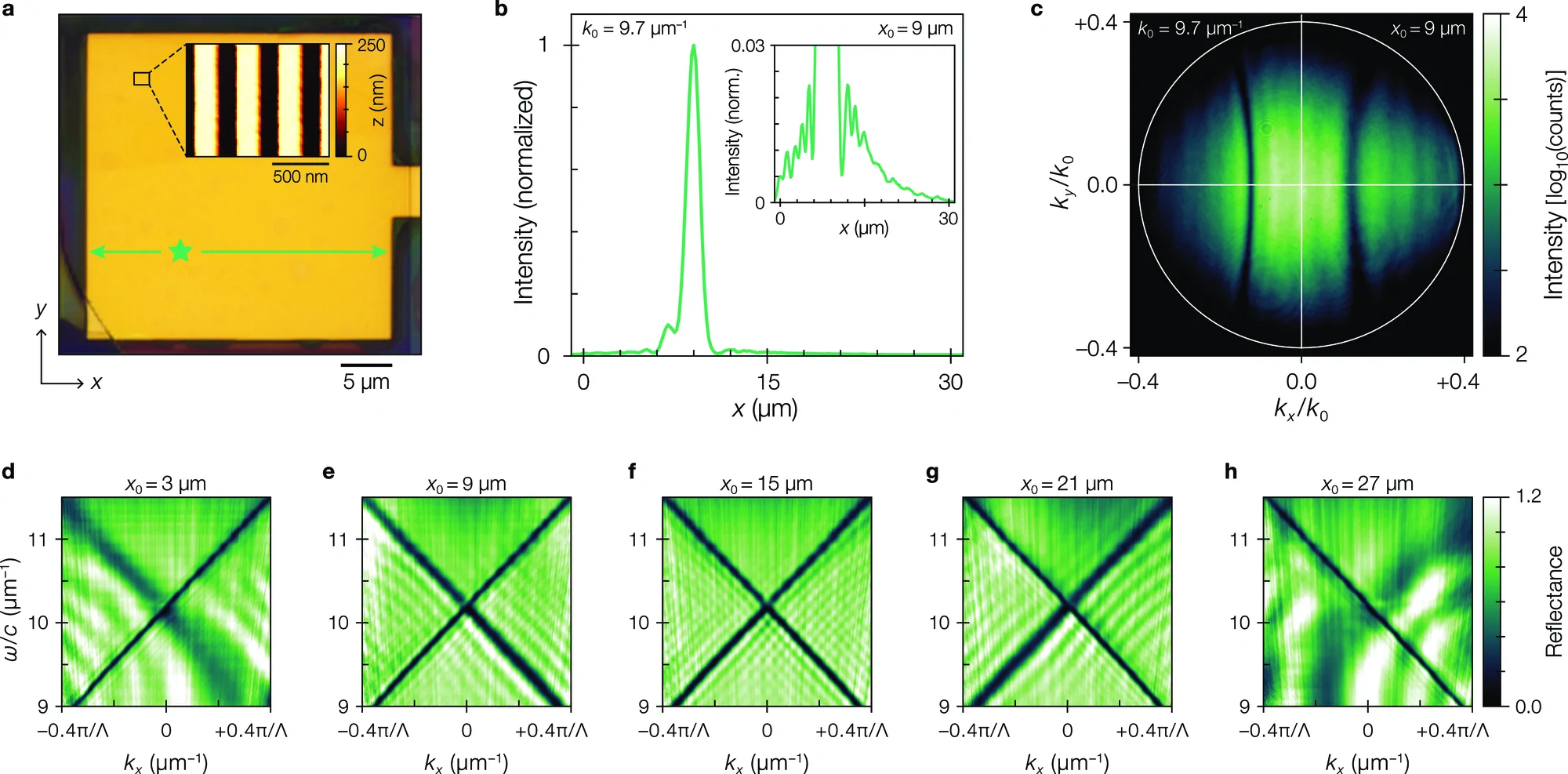
Position-dependent line width and interference pattern. (a) Optical micrograph of the fabricated resonator. Inset: atomic force micrograph of the subwavelength grating. The green star marks the excitation position (*x*_0_ = 9 μm). (b) Horizontal linecut through the center of the reflected beam imaged in real-space. Inset: same image recorded with a longer exposure to reveal the low-intensity features, scaled to match the intensity in the main panel. (c) Corresponding reflection imaged in momentum space (Fourier plane). (d–h) Measured momentum-resolved reflectance at different incident positions *x*_0_, confirming the predicted interference fringes and line width variation.

We mount the completed sample in a diffraction-limited optical microscope and illuminate it with a focused monochromatic laser beam (20× objective, NA = 0.4) at *k*_0_ = 9.7 μm^–1^, centered at *x*_0_ = 9 μm. In contrast to the STCMT model, the symmetry of the grating leads to the excitation of two counter-propagating guided waves from the illumination spot. Figure 4b shows a horizontal linecut through the real-space reflected intensity profile imaged on the camera. When recorded with a longer exposure time, a delocalized radiation pattern and exponential decay away from *x*_0_ become apparent, characteristic of a leaky guided resonance (inset of Figure 4b).

By inserting a Bertrand lens, we image the back-focal (Fourier) plane of the microscope objective to obtain the scattered field in momentum space (Figure 4c). Two resonant features are observed as dips in the reflectance at *k*_*x*_/*k*_0_ ≈ ± 0.13. Because the excitation is off-center, the left- and right-propagating guided waves experience different interaction lengths. From the phase-matching condition of the forward mode, *k*_*x*_ + *q* = β, we obtain *k*_*x*_/*k*_0_ = −0.13 and we therefore identify the feature at negative *k*_*x*_ as right-propagating. This assessment is directly corroborated by the back-focal plane image, where this branch exhibits a narrower resonance than the branch at *k*_*x*_/*k*_0_ = +0.13, owing to its longer interaction length *L*.

To study the position dependence of the resonant lineshapes in more detail, we scan the laser spot over the metasurface from *x* = 0 μm to *x* = *W* = 30 μm in 1 μm steps. At each position, we sweep the laser frequency and record the momentum-resolved reflectance. Figure 4d–h shows the measured dispersion at five representative incident positions. When the metasurface is illuminated near its center (Figure 4f), the response is symmetric and both counter-propagating modes exhibit a narrow resonance while already showing an interference pattern indicating that the finite sample size affects the response. As the spot is moved toward the left (Figure 4e), this symmetry is broken: the right-propagating branch narrows (due to a longer *L*) while the left-propagating branch broadens, eventually becoming barely discernible near the edge (Figure 4d). This trend reverses when moving to the right edge (Figure 4g,h), consistent with the swapped interaction lengths experienced by the two guided waves. To further illustrate this modal evolution, we plot the position-dependent angular reflectance at a fixed *k*_0_ = 9.7 μm^–1^ (Figure 5a), emphasizing how both the resonance line shape and the interference fringes vary with the excitation position. Overall, these observations are in excellent agreement with the STCMT predictions (Figure 3).

**Figure 5:**
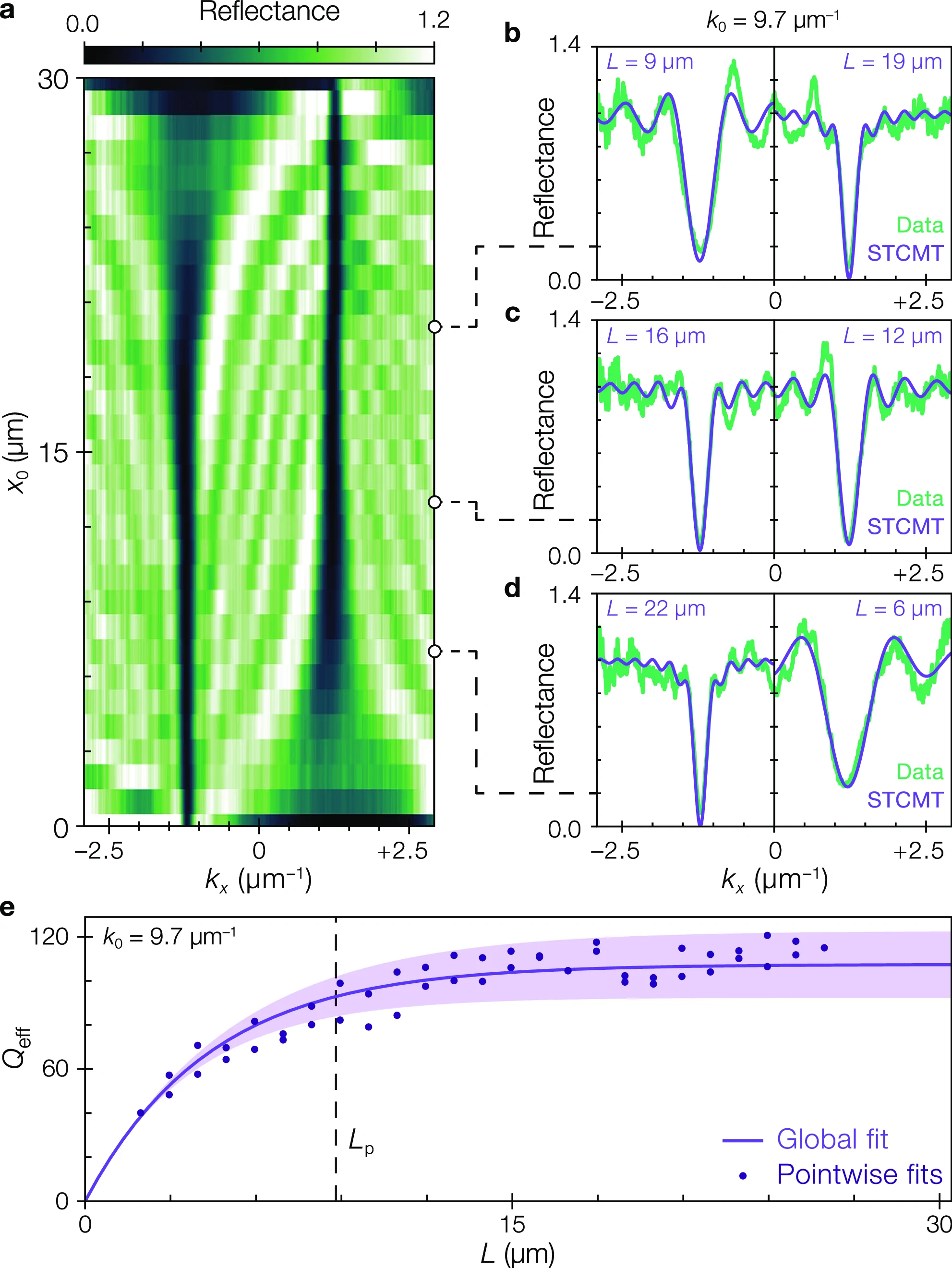
Comparison between model and experiment. (a) Measured reflectance versus excitation position *x*_0_ and transverse momentum *k*_*x*_, at *k*_0_ = 9.7 μm^–1^. (b–d) Representative momentum-resolved reflectance linecuts (green) at selected *x*_0_, overlaid with the global STCMT fit (purple). The fitted lengths *L* are indicated. (e) Quality factor *Q*_eff_(*L*) inferred from the global STCMT fit, with the extracted propagation length *L*_p_ indicated (dashed line). Points represent fits performed independently at each linecut; the shaded band indicates the corresponding model discrepancy (one standard deviation) relative to these pointwise fits.

Motivated by this qualitative correspondence, we next extract the intrinsic decay rates, Γ_int_ and Γ_ext_, by fitting the measured reflectance, while fixing β and *v*_*g*_ from the eigenmode dispersion to reduce the parameter space (Figure 2b). To capture experimental deviations, we introduce Δ*k*_*x*_ to describe a small systematic offset in the phase-matching condition, and Δ*L* to account for a reduced effective interaction length (scattering aperture). The latter may arise from a nonabrupt or displaced optical boundary in the fabricated metasurface, particularly because the grating and gold back-reflector do not terminate at exactly the same position. Figure 5b–d shows representative linecuts at three excitation positions *x*_0_, with the STCMT fitted separately to the left- and right-propagating branches (since the STCMT was developed for a single mode only). The model accurately reproduces the reflectance data across the scanned range for Δ*k*_*x*_ ≈ 0.08 μm^–1^ and Δ*L* ≈ −1.05 μm, including many of the smaller fringes around the resonant dips. From these fits, we obtain Γ_ext_ ≈ 7.1 × 10^12^ s^–1^ and Γ_int_ ≈ 6.5 × 10^12^ s^–1^, corresponding to a propagation length *L*_p_ ≈ 8.8 μm. Via eq 11, we then retrieve the experimental *Q*_eff_(*L*) curve with *Q*_eff_ → 107 as *L* ≫ *L*_p_ (Figure 5e). Although the extracted radiative rate agrees closely with the modeled value Γ_ext_^sim^ = 7.4 × 10^12^ s^–1^, the nonradiative rate exceeds the modeled Γ_int_^sim^ = 3.9 × 10^12^ s^–1^ by almost a factor of 2, which we attribute to additional dissipation due to surface roughness, polymer residues, and spatial inhomogeneity. As a result, the experimental *L*_p_ and *Q* are smaller than the modeled values (Figure 3c). To nevertheless assess the model discrepancy, we refit *Q*_eff_ independently at each *x*_0_ (data points in Figure 5e) and use this to define an uncertainty range on *Q*_eff_ (shaded region). These pointwise fits reproduce the same overall trend, further supporting the validity of our model.

### Design Principles

Although this work focused on a specific guided-mode resonant metasurface with a moderate *Q*-factor to demonstrate the impact of finite size, we stress that our observations apply more broadly. In principle, any propagating nonlocal mode that can be described by a locally linear dispersion will feature a *Q*-factor approaching the semi-infinite limit as 1–*e* ^–2*L*/*L*_p_^. Concretely, our design advice for reaching the long-grating regime, in which finite-size effects can mostly be neglected, is to fabricate a metasurface with a physical width of 5× the modal propagation length. This ensures that a mode excited in the center of the structure (*L*/*L*_p_ = 2.5) decays to about 8% of its original amplitude and the effective *Q*-factor reaches 99.3% of the theoretical value. Of course, it may be interesting to operate in the limit of a size-constrained grating. Remarkably, in this regime, the quality factor is primarily governed by the mode’s transit time to the edge (Supporting Information Section S4). This implies that, for strongly footprint-constrained devices, high-*Q* requires increasing the interaction time within the fixed footprint by engineering flat bands, i.e., slow light, provided that intrinsic dissipative losses remain sufficiently low.^41,68^

Another aspect that we have not examined so far is the impact of the NA (spot size) of the driving field, which relates to the experimental design rather than the sample size. In general, for an infinite grating, it is favorable to minimize the angular bandwidth such that all of the incident power is concentrated at *k*_*x*,res_. This can be particularly important for applications requiring the highest possible light-matter interactions, such as nonlinear optics.

In the following, we consider a finite-NA sinc-beam to explore how the stored energy *U* in the resonator is maximized by matching the spot size to the grating width (details in Supporting Information Section S4). Keeping the total incident power fixed at an arbitrary value of *P*_in_ = 1*W*, we vary the NA to control the diffraction-limited spot size. We then evaluate

12 $$U\equiv \int_{0}^{W}|a\left(x\right){|}^{2}dx$$

by plugging the solution of the mode amplitude, eq 9 for various normalized widths *W*/*L*_p_ (Figure 6a), observing a pronounced maximum that is captured well by the simple coherent aperture-overlap,

13 $$\int w\left(x\right)s_{+}\left(x\right)dx$$

Note that *U* does not vary monotonically with NA, but rather features a maximum at some optimal NA value. For a sinc-beam centered on the grating (*L* = *W*/2), we find the optimum to be NA_opt_ ≈ 0.685 λ_0_/*W*. While the numerical prefactor depends on the specific excitation conditions assumed here, the overall scaling NA_opt_ ∝ λ_0_/*W* holds generally.

**Figure 6:**
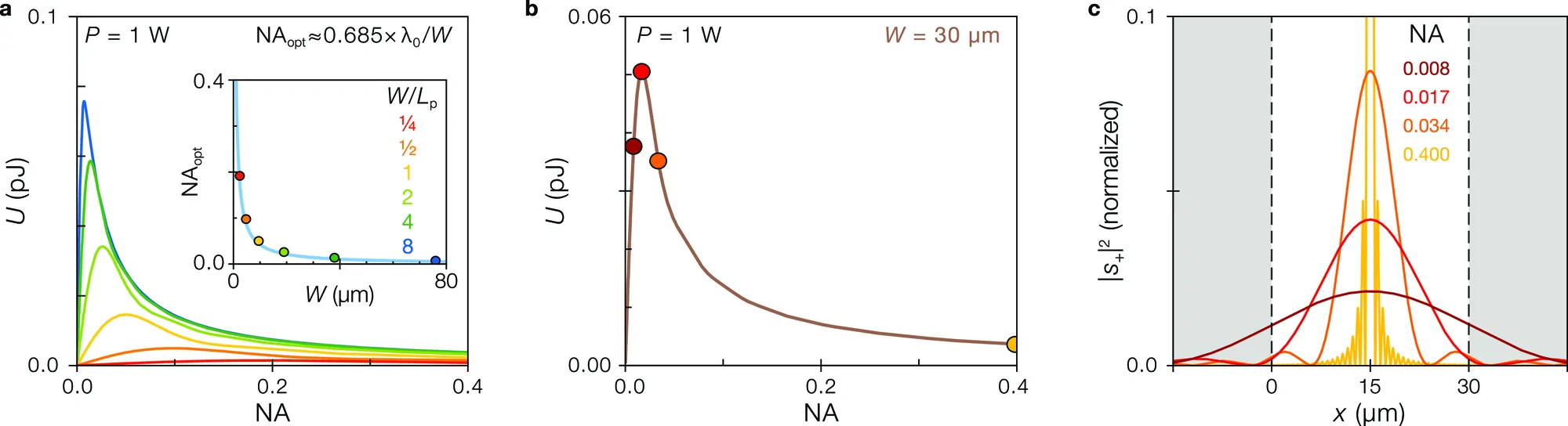
Maximizing stored energy by matching illumination width to the grating. (a) Stored energy (*U*) as a function of incident NA, calculated for varying grating widths *W*/*L*_p_ (color) under sinc excitation in the center (*L* = *W*/2) with constant total incident power *P* = 1 *W*. Inset: the optimal NA extracted at each width (colored data points) is well approximated by NA_opt_ ≈ 0.685 λ_0_/*W* (light blue curve). (b) Stored energy versus NA (brown curve) for the *W* = 30 μm grating discussed in the main text, with four highlighted data points corresponding to (c) the normalized incident intensity profiles |*s*_+_(*x*)|^2^ for the same four NA values. The grating boundaries are indicated (dashed lines); incident field outside the grating (gray shaded region) does not contribute to resonant energy build-up.

To explore the origin of this maximum, let us again consider the 30-μm-wide metasurface discussed throughout this work (Figure 6b). We choose four representative numerical apertures to show that for a finite grating, as NA → 0 the illumination profile *s*_+_(*x*) starts to extend well beyond the grating window [0, *W*], and an increasing fraction of the incident power cannot excite the resonant mode (Figure 6c). On the other hand, if the NA increases, an increasing fraction of the incident power is at wavenumbers outside of the resonant wavenumber, increasing reflection and reducing power coupled into the resonance. As a result, *U* is maximized when the spot size is matched to the grating width, or vice versa, reflecting the trade-off between matching the real-space footprint and matching the resonance in momentum space.

In sum, we have formalized the relations among the modal propagation length, physical metasurface footprint, excitation spot size, and critical performance metrics of nonlocal metasurfaces, which is especially relevant amid the recent push toward ultrahigh *Q*-factors. Our work provides practicable design guidelines that can be leveraged to reach the theoretical line width and maximize the light-matter interaction: (i) to achieve a *Q*-factor close to that of the infinite array, the metasurface needs to be over five propagation lengths long, and (ii) to maximize the stored power in the resonator, the array size must be matched to the central lobe of the incident beam.

## Discussion and Conclusions

We present the effects of a finite lateral extent on the optical properties of leaky-wave metasurfaces. In particular, we leverage the STCMT framework to derive approximate analytic expressions for the spectrospatial scattering response of a guided-mode resonator with a truncated grating. This reveals that, when the interaction length is on the order of or shorter than the guided wave’s propagation length, the scattered field shows strong interference fringes that can exceed unity reflectance due to a redistribution of spectral weight in momentum space, as well as a reduction of the effective lifetime and therefore the energy stored in the resonator. To validate these results, we fabricated the described metasurface and performed position- and momentum-resolved reflectance spectroscopy. These measurements demonstrate the finite-size effects, observing an excellent agreement between theory and experiment. More generally, our model offers a straightforward method to predict the maximum achievable quality factor in a limited footprint given the theoretical dispersion of the guided wave. This further establishes STCMT as a valuable technique for both qualitatively and quantitatively capturing the scattering response of real-world nonlocal meta-optical elements.

Our results are directly relevant to emerging applications, including resonant spaceplates, where a reduction in the quality factor may limit the achievable compression ratio, while finite-aperture interference could distort the complex-amplitude response required to emulate free-space propagation.^69,70^ More broadly, the versatility of STCMT leaves many promising avenues for further inquiry, such as the use of in-plane reflectors to increase the effective interaction length by trapping light within a finite scattering aperture.^56,57^ Other directions include metasurfaces exhibiting quadratic dispersion arising from quasi-bound states in the continuum or strongly coupled counter-propagating leaky waves, as well as two-port systems for modeling transmission-based meta-optics.^9,31,41,61^ Altogether, the framework presented here provides fundamental insights into the impact of finite size on the resonant response of these emerging photonic systems—an essential step toward technological integration in augmented reality, biosensing, and nonlinear optics.

## Supplementary Material



## Data Availability

A full replication package including all data and scripts is available at 10.6084/m9.figshare.33268896.

## Notes

Sample fabrication, optical measurement, and numerical simulation methods; dispersion relation; derivations of the spatiotemporal coupled-mode equations and reflection coefficient; assessment of the point-excitation approximation; derivations of resonant antireflection, stored energy, edge power flux, quality factor, and interference fringes (PDF)

## References

[1] Yu N., Capasso F. (2014). Flat Optics with Designer Metasurfaces. Nat. Mater..

[2] Kuznetsov A. I., Brongersma M. L., Yao J. (2024). Roadmap for Optical
Metasurfaces. ACS Photonics.

[3] Bandiera S., Jacob D., Muller T., Marquier F., Laroche M., Greffet J.-J. (2008). Enhanced Absorption
by Nanostructured Silicon. Appl. Phys. Lett..

[4] Kim S. J., Brongersma M. L. (2017). Active Flat Optics Using a Guided Mode Resonance. Opt. Lett..

[5] Lawrence M., Barton D. R., Dixon J., Song J.-H., Van De
Groep J., Brongersma M. L., Dionne J. A. (2020). High Quality Factor
Phase Gradient Metasurfaces. Nat. Nanotechnol..

[6] Hsu C. W., Zhen B., Lee J., Chua S.-L., Johnson S. G., Joannopoulos J. D., Soljačić M. (2013). Observation of Trapped
Light within the Radiation Continuum. Nature.

[7] Koshelev K., Lepeshov S., Liu M., Bogdanov A., Kivshar Y. (2018). Asymmetric
Metasurfaces with High-$Q$ Resonances Governed by Bound States in
the Continuum. Phys. Rev. Lett..

[8] Jin J., Yin X., Ni L., Soljačić M., Zhen B., Peng C. (2019). Topologically
Enabled Ultrahigh-Q Guided Resonances Robust to Out-of-Plane
Scattering. Nature.

[9] Shastri K., Monticone F. (2023). Nonlocal Flat
Optics. Nat. Photonics.

[10] Chen Z., Yin X., Jin J., Zheng Z., Zhang Z., Wang F., He L., Zhen B., Peng C. (2022). Observation of Miniaturized Bound
States in the Continuum with Ultra-High Quality Factors. Sci. Bull..

[11] Huang L., Jin R., Zhou C., Li G., Xu L., Overvig A., Deng F., Chen X., Lu W., Alù A., Miroshnichenko A. E. (2023). Ultrahigh-Q Guided Mode Resonances
in an All-dielectric
Metasurface. Nat. Commun..

[12] Fang J., Chen R., Sharp D. (2024). Million-Q
Free Space
Meta-Optical Resonator at Near-Visible Wavelengths. Nat. Commun..

[13] Overvig A. C., Malek S. C., Yu N. (2020). Multifunctional
Nonlocal Metasurfaces. Phys. Rev. Lett..

[14] Overvig A., Alù A. (2022). Diffractive
Nonlocal Metasurfaces. Laser Photonics Rev..

[15] Chai R., Liu Q., Liu W., Li Z., Cheng H., Tian J., Chen S. (2023). Emerging Planar Nanostructures
Involving Both Local and Nonlocal
Modes. ACS Photonics.

[16] Wang J., Kühne J., Karamanos T., Rockstuhl C., Maier S. A., Tittl A. (2021). All-Dielectric
Crescent Metasurface
Sensor Driven by Bound States in the Continuum. Adv. Funct. Mater..

[17] Kühner L., Sortino L., Berté R., Wang J., Ren H., Maier S. A., Kivshar Y., Tittl A. (2022). Radial Bound States
in the Continuum for Polarization-Invariant Nanophotonics. Nat. Commun..

[18] Hu J., Safir F., Chang K., Dagli S., Balch H. B., Abendroth J. M., Dixon J., Moradifar P., Dolia V., Sahoo M. K., Pinsky B. A., Jeffrey S. S., Lawrence M., Dionne J. A. (2023). Rapid Genetic
Screening with High
Quality Factor Metasurfaces. Nat. Commun..

[19] Kodigala A., Lepetit T., Gu Q., Bahari B., Fainman Y., Kanté B. (2017). Lasing Action
from Photonic Bound States in Continuum. Nature.

[20] Ha S. T., Fu Y. H., Emani N. K., Pan Z., Bakker R. M., Paniagua-Domínguez R., Kuznetsov A. I. (2018). Directional
Lasing in Resonant Semiconductor Nanoantenna Arrays. Nat. Nanotechnol..

[21] Hwang M.-S., Lee H.-C., Kim K.-H., Jeong K.-Y., Kwon S.-H., Koshelev K., Kivshar Y., Park H.-G. (2021). Ultralow-Threshold
Laser Using Super-Bound States in the Continuum. Nat. Commun..

[22] Benea-Chelmus I. C., Mason S., Meretska M. L., Elder D. L., Kazakov D., Shams-Ansari A., Dalton L. R., Capasso F. (2022). Gigahertz Free-Space
Electro-Optic Modulators Based on Mie Resonances. Nat. Commun..

[23] Damgaard-Carstensen C., Yezekyan T., Brongersma M. L., Bozhevolnyi S. I. (2025). Highly
Efficient, Tunable, Electro-Optic, Reflective Metasurfaces Based on
Quasi-Bound States in the Continuum. ACS Nano.

[24] Hoekstra T., van de Groep J. (2026). Electrically
Tunable Strong Coupling in a Hybrid-2D
Excitonic Metasurface for Optical Modulation. Light:Sci. Appl..

[25] Song J. H., van de Groep J., Kim S. J., Brongersma M. L. (2021). Non-Local
Metasurfaces for Spectrally Decoupled Wavefront Manipulation and Eye
Tracking. Nat. Nanotechnol..

[26] Malek S. C., Overvig A. C., Alù A., Yu N. (2022). Multifunctional Resonant
Wavefront-Shaping Meta-Optics Based on Multilayer and Multi-Perturbation
Nonlocal Metasurfaces. Light:Sci. Appl..

[27] Koshelev K., Tang Y., Li K., Choi D.-Y., Li G., Kivshar Y. (2019). Nonlinear Metasurfaces
Governed by Bound States in
the Continuum. ACS Photonics.

[28] Liu Z., Xu Y., Lin Y., Xiang J., Feng T., Cao Q., Li J., Lan S., Liu J. (2019). High-Q Quasibound States in the Continuum
for Nonlinear Metasurfaces. Phys. Rev. Lett..

[29] Zograf G., Koshelev K., Zalogina A., Korolev V., Hollinger R., Choi D.-Y., Zuerch M., Spielmann C., Luther-Davies B., Kartashov D., Makarov S. V., Kruk S. S., Kivshar Y. (2022). High-Harmonic Generation
from Resonant Dielectric Metasurfaces
Empowered by Bound States in the Continuum. ACS Photonics.

[30] Kwon H., Sounas D., Cordaro A., Polman A., Alù A. (2018). Nonlocal Metasurfaces
for Optical Signal Processing. Phys. Rev. Lett..

[31] Cordaro A., Kwon H., Sounas D., Koenderink A. F., Alù A., Polman A. (2019). High-Index Dielectric
Metasurfaces
Performing Mathematical Operations. Nano Lett..

[32] Overvig A. C., Mann S. A., Alù A. (2021). Thermal Metasurfaces:
Complete Emission
Control by Combining Local and Nonlocal Light-Matter Interactions. Phys. Rev. X.

[33] De
Luca F., Guddala S., Cotrufo M., Touma J., Overvig A., Alù A. (2025). Nonlocal Metasurface Lens for Long-Wavelength Infrared
Radiation. Adv. Mater..

[34] Klopfer E., Dagli S., Barton D. I., Lawrence M., Dionne J. A. (2022). High-Quality-Factor
Silicon-on-Lithium Niobate Metasurfaces for Electro-optically Reconfigurable
Wavefront Shaping. Nano Lett..

[35] Lin L., Hu J., Dagli S., Dionne J. A., Lawrence M. (2023). Universal Narrowband
Wavefront Shaping with High Quality Factor Meta-Reflect-Arrays. Nano Lett..

[36] Hail C. U., Foley M., Sokhoyan R., Michaeli L., Atwater H. A. (2023). High Quality
Factor Metasurfaces for Two-Dimensional Wavefront Manipulation. Nat. Commun..

[37] Rodriguez S. R. K., Schaafsma M. C., Berrier A., Gómez Rivas J. (2012). Collective
Resonances in Plasmonic Crystals: Size Matters. Phys. B.

[38] Grepstad J. O., Greve M. M., Holst B., Johansen I.-R., Solgaard O., Sudbø A. (2013). Finite-Size Limitations on Quality Factor of Guided
Resonance Modes in 2D Photonic Crystals. Opt.
Express.

[39] Yang Y., Kravchenko I. I., Briggs D. P., Valentine J. (2014). All-Dielectric
Metasurface Analogue of Electromagnetically Induced Transparency. Nat. Commun..

[40] Zundel L., Manjavacas A. (2019). Finite-Size Effects on Periodic Arrays of Nanostructures. J. Phys.: Photonics.

[41] Taghizadeh A., Chung I.-S. (2017). Quasi Bound States in the Continuum
with Few Unit Cells
of Photonic Crystal Slab. Appl. Phys. Lett..

[42] Droulias S., Koschny T., Soukoulis C. M. (2018). Finite-Size Effects in Metasurface
Lasers Based on Resonant Dark States. ACS Photonics.

[43] Wang S. S., Moharam M. G., Magnusson R., Bagby J. S. (1990). Guided-Mode Resonances
in Planar Dielectric-Layer Diffraction Gratings. J. Opt. Soc. Am. A.

[44] Wang S. S., Magnusson R. (1993). Theory and Applications of Guided-Mode Resonance Filters. Appl. Opt..

[45] Brazas J. C., Li L. (1995). Analysis of Input-Grating Couplers Having Finite Lengths. Appl. Opt..

[46] Saarinen J., Noponen E., Turunen J. P. (1995). Guided-Mode Resonance Filters of
Finite Aperture. Opt. Eng..

[47] Boye R. R., Kostuk R. K. (2000). Investigation of
the Effect of Finite Grating Size
on the Performance of Guided-Mode Resonance Filters. Appl. Opt..

[48] Jacob D. K., Dunn S. C., Moharam M. G. (2000). Design Considerations for Narrow-Band
Dielectric Resonant Grating Reflection Filters of Finite Length. J. Opt. Soc. Am. A.

[49] Bendickson J. M., Glytsis E. N., Gaylord T. K., Brundrett D. L. (2001). Guided-Mode
Resonant Subwavelength Gratings: Effects of Finite Beams and Finite
Gratings. J. Opt. Soc. Am. A.

[50] Jeong D.-Y., Ye Y. H., Zhang Q. M. (2002). Effective
Optical Properties Associated
with Wave Propagation in Photonic Crystals of Finite Length along
the Propagation Direction. J. Appl. Phys..

[51] Peters D. W., Kemme S. A., Hadley G. R. (2004). Effect
of Finite Grating, Waveguide
Width, and End-Facet Geometry on Resonant Subwavelength Grating Reflectivity. J. Opt. Soc. Am. A.

[52] Block I. D., Mathias P. C., Jones S. I., Vodkin L. O., Cunningham B. T. (2009). Optimizing
the spatial resolution of photonic crystal label-free imaging. Appl. Opt..

[53] Triggs G. J., Fischer M., Stellinga D., Scullion M. G., Evans G. J. O., Krauss T. F. (2015). Spatial Resolution and Refractive Index Contrast of
Resonant Photonic Crystal Surfaces for Biosensing. IEEE Photonics J..

[54] Haus, H. A. Waves and fields in optoelectronics; Prentice-Hall series in solid state physical electronics; Prentice-Hall: Englewood Cliffs, NJ, 1984.

[55] Fan S., Suh W., Joannopoulos J. D. (2003). Temporal coupled-mode theory for
the Fano resonance in optical resonators. J.
Opt. Soc. Am. A.

[56] Kintaka K., Majima T., Inoue J., Hatanaka K., Nishii J., Ura S. (2012). Cavity-Resonator-Integrated Guided-Mode Resonance Filter for Aperture
Miniaturization. Opt. Express.

[57] Dolia V., Balch H. B., Dagli S., Abdollahramezani S., Carr Delgado H., Moradifar P., Chang K., Stiber A., Safir F., Lawrence M., Hu J., Dionne J. A. (2024). Very-Large-Scale-Integrated
High Quality Factor Nanoantenna Pixels. Nat.
Nanotechnol..

[58] Hao R., Ye B., Xu J., Zou Y. (2025). Performance Optimization of Planar
Photonic Crystal Bound States in the Continuum Cavities: Mitigating
Finite-Size Effects. Front. Optoelectron..

[59] Ustimenko N., Rockstuhl C., Evlyukhin A. B. (2024). Resonances
in Finite-Size All-Dielectric
Metasurfaces for Light Trapping and Propagation Control. Phys. Rev. B.

[60] Hoang T. X., Leykam D., Chu H.-S., Png C. E., García-Vidal F. J., Kivshar Y. S. (2025). Collective Nature
of High-Q Resonances in Finite-Size
Photonic Metastructures. Phys. Rev. Res..

[61] Bykov D. A., Doskolovich L. L. (2015). Spatiotemporal
Coupled-Mode Theory of Guided-Mode Resonant
Gratings. Opt. Express.

[62] Overvig A., Mann S. A., Alù A. (2024). Spatio-Temporal
Coupled Mode Theory
for Nonlocal Metasurfaces. Light:Sci. Appl..

[63] Jeon D., Kim H., Rho J. (2025). Leaky Guided Mode-Induced
Large-Angle Nonlocal Metasurfaces. ACS Photonics.

[64] Yakubovsky D. I., Arsenin A. V., Stebunov Y. V., Fedyanin D. Y., Volkov V. S. (2017). Optical
constants and structural properties of thin gold films. Opt. Express.

[65] Mann S. A., Sounas D. L., Alù A. (2019). Nonreciprocal cavities and the time-bandwidth
limit. Optica.

[66] Castellanos-Gomez A., Buscema M., Molenaar R., Singh V., Janssen L., Van Der Zant H. S., Steele G. A. (2014). Deterministic Transfer of Two-Dimensional
Materials by All-Dry Viscoelastic Stamping. 2D Mater..

[67] Shen F., Liu D., Chen Z., Zhu J., Jin S., Zhao X., Ma Y., Lei D., Xu J. (2025). Manipulating the Intrinsic Light–Matter
Interaction with High-Q Resonances in an Etch-Free van Der Waals Metasurface. Optica.

[68] Barton D., Hu J., Dixon J., Klopfer E., Dagli S., Lawrence M., Dionne J. (2021). High-Q Nanophotonics: Sculpting Wavefronts with Slow
Light. Nanophotonics.

[69] Guo C., Wang H., Fan S. (2020). Squeeze Free
Space with Nonlocal
Flat Optics. Optica.

[70] Shastri K., Reshef O., Boyd R. W., Lundeen J. S., Monticone F. (2022). To What Extent
Can Space Be Compressed? Bandwidth Limits of Spaceplates. Optica.

