# Supplementary material for: Finite-Size Effects in Nonlocal Metasurfaces
Source: ACS Photonics. 2026 Sep 2;13(18):5337–46. doi: 10.1021/acsphotonics.6c00860 (PMC13587770; doi:10.1021/acsphotonics.6c00860)
Supplement: Supplementary file 1 [file ph6c00860_si_001.pdf]

Supporting Information for: Finite-Size Effects in Nonlocal Metasurfaces

Tom Hoekstra[1], Sander A. Mann[1], and Jorik van de Groep*[1]

[1]Van der Waals-Zeeman Institute, Institute of Physics, University of Amsterdam, 1098 XH, Amsterdam, the Netherlands

*Email: j.vandegroep@uva.nl

This file includes:

- Methods
- Sections S1–4
- Figures S1–3

Methods

Sample fabrication

A bulk hBN crystal (HQ Graphene) is mechanically exfoliated using Nitto SPV-224 tape onto stamps cut from polydimethylsiloxane sheets (Gel-Pak WF-30). The stamps are inspected in a confocal microscope (WITec α-300) and hBN flakes of suitable thickness are identified using white-light reflectometry. The target substrate, with the prepatterned Au back-reflector, is mounted in a custom-built stamping microscope equipped with a temperature-controlled stage. After aligning the chosen hBN flake over the back-reflector region, the stamp is brought into contact with the substrate and held at 60 °C for 5 min. The stamp is subsequently retracted at 0.1 µm/s, leaving the hBN flake on the substrate. To improve adhesion and reduce surface contamination, the sample is annealed in a vacuum tube furnace ($P \approx 10^{-6}$–10^{-7} mbar) at 150 °C for 8 h. The surface is further cleaned with an O_2 plasma descum for 30 s (Oxford Instruments Plasma 80). A 222 nm layer of CSAR AR-P 62 e-beam resist is spin-coated and baked at 150 °C for 120 s. The grating is patterned by electron-beam lithography (Raith Voyager) using a dose of 130.8 µC/cm^2. Development is performed in n-amyl acetate for 60 s, followed by sequential rinses in o-xylene (7 s) and MIBK:IPA 9:1 (15 s). The sample is finally rinsed in IPA and dried under N_2. Atomic force microscopy (Bruker Dimension FastScan) is performed to verify the fabricated grating dimensions against the design.

Optical measurements

Reflection measurements are performed with a diffraction-limited confocal microscope (WITec α-300). As the light source, we use an NKT Photonics SuperK tunable laser system coupled into the microscope via a single-mode photonic crystal fiber (Connect FD7-PM). Linearly polarized illumination is provided by a pulsed supercontinuum source (Extreme EXW-12; 78 MHz repetition rate, ∼5 ps pulse width), which is spectrally selected with an acousto-optic tunable filter (Select VIS/1X) to yield coherent monochromatic light with a bandwidth of ∼1 nm across the visible range. The collimated beam is directed to the entrance pupil (Fourier plane) of the objective (Zeiss EC Epiplan, 20×, NA = 0.4), producing a diffraction-limited spot at the sample plane. The sample is mounted on the microscope piezo stage for precise lateral positioning.

The reflected signal is collected by the same objective and imaged onto a 14-bit CMOS camera (Zeiss Axiocam 705 mono). A Bertrand lens can be inserted in the detection path to switch between real-space and momentum-space imaging. The incident power and camera exposure time are calibrated and adjusted slightly per wavelength to maximize the signal-to-noise ratio; the incident power is in the range 36–80 nW. For the position-dependent dispersion measurements, we automate wavelength sweeps and piezo-stage scans using a custom Python control script. Two reference measurements are acquired: one without a sample mounted to account for internal reflections, and one on an exposed gold region for normalization. The latter uses the theoretical angle- and wavelength-dependent reflectance of thin-film Au calculated from tabulated permittivity data.[1]

Numerical simulations

The field distribution and reflectance dispersion of the infinite structure are simulated using S^4 rigorous coupled-wave analysis.[2] The eigenmode dispersion is calculated with a custom slab waveguide mode solver, in which the grating is treated as an effective medium. For hBN and Au, we use tabulated optical constants,[1,3] whereas for CSAR we extract the permittivity from spectroscopic ellipsometry measurements (J.A. Woollam VB-400). The spatiotemporal coupled-mode equations are derived analytically, with full details provided in Supplementary Sections S1, S2 and S3. All simulations are carried out using custom Python scripts.

S1 Dispersion relation

Consider a quasi-guided mode propagating in the positive x-direction described by the modal parameter $a(x,t)$, which evolves as

$$a(x,t) \propto e^{i\beta x - i\omega t} \tag{1}$$

with phase constant β and frequency ω. Throughout, we approximate the dispersion as locally linear (Fig. S1), by expanding the complex propagation constant to first-order near a reference point (β_0,ω_0) as

$$\tilde{\beta}(\omega) = \beta + i\alpha \simeq \beta_0 + \frac{\omega - \omega_0}{v_g} + i\alpha, \qquad v_g \equiv \left.\frac{d\omega}{d\beta}\right|_{\beta_0}, \tag{2}$$

where α is the spatial attenuation rate. Equivalently, in the momentum-domain picture we can write the complex frequency

$$\Omega(\beta) \equiv \omega - i\Gamma \simeq \omega_0 - i\Gamma + v_g(\beta - \beta_0), \tag{3}$$

where $\Gamma \equiv \Gamma_{\text{ext}} + \Gamma_{\text{int}} = v_g\alpha$ is the temporal decay rate comprising the external (radiative) decay rate Γ_{ext} associated with coupling of the guided mode to the far field via the grating, and the intrinsic, non-radiative loss rate Γ_{int}. A closely related parameter is the modal propagation length,

$$L_p = \frac{1}{\alpha} = \frac{v_g}{\Gamma}, \tag{4}$$

i.e., the distance over which the mode amplitude decays to $1/e$. This parameter is particularly important because it governs the characteristic length scale over which finite-size effects cannot be ignored.

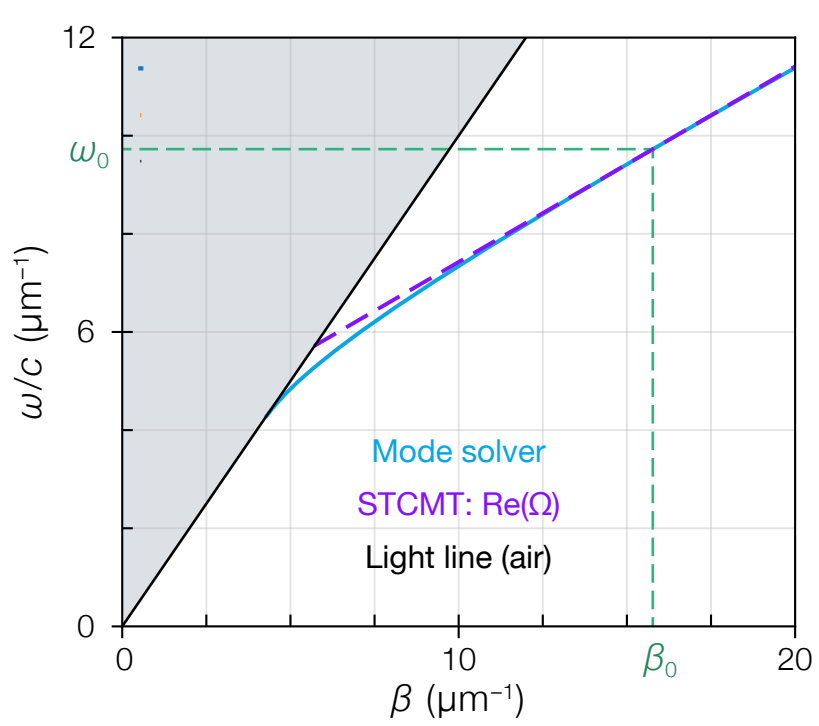

Figure S1: Locally linear complex dispersion relation. Simulated TE_0 eigenmode dispersion obtained with a slab waveguide mode solver by treating the grating as an effective medium (blue). Overlaid is the approximate STCMT dispersion Ω (purple, dashed) which is linearized around the point ω_0, β_0 (green, dashed). For reference, the light cone (shaded gray) and light line in air (black) are shown.

To parametrize the dispersion within STCMT, *i.e.* Eq. (3), we obtain the guided mode dispersion numerically using a slab waveguide mode solver by modeling the grating as an effective medium. However, since the eigenmode is computed for a closed system, we must obtain the radiative rate Γ_{ext} by other means. In this case, we use full-wave simulations to model the driven response of the semi-infinite metasurface and fitting it with the well-known coupled-mode theory expression for a single mode with one external port (*i.e.* Eq. (46)).[4–6] From this analysis, we find that a constant value of $\Gamma_{\text{ext}} = 7.4 \times 10^{12}$ s^{-1} is sufficient to accurately reproduce the full-wave response over the bandwidth considered here (Fig. S2). The observed deviation at higher frequencies is due to increasing optical losses in the gold layer, which is not accounted for in our model (constant background amplitude $C = -1$).

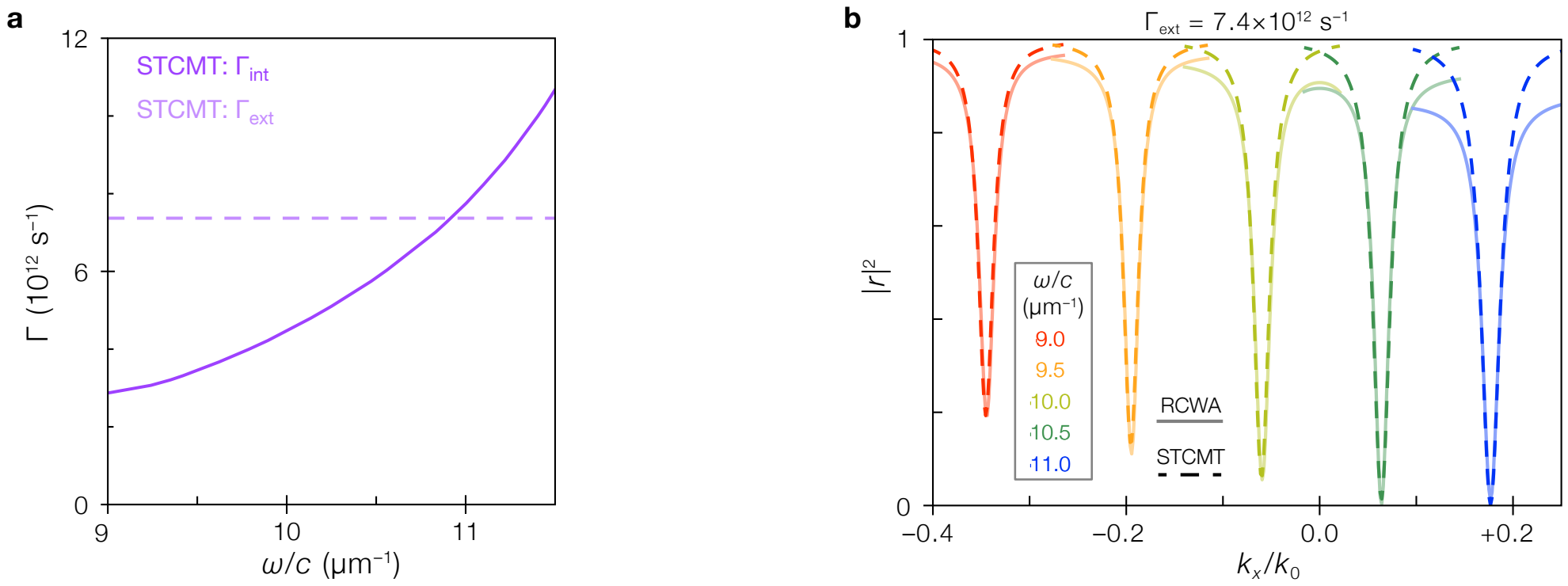

Figure S2: Comparison with full-wave simulations. **(a)** Loss rates used in STCMT: the eigenmode-solver decay rate is taken as the internal (non-radiative) loss rate Γ_{int} (purple, solid), while the external (radiative) loss rate Γ_{ext} is treated as approximately constant over the relevant frequency band and extracted by fitting to full-wave simulations (pink, dashed; $\Gamma_{\text{ext}} = 7.4 \times 10^{12}\,\text{s}^{-1}$). **(b)** Momentum-resolved reflectance $|r(k_x)|^2$ from full-wave RCWA at selected frequencies (solid), overlaid with the corresponding STCMT prediction (dashed) evaluated in the long-grating limit. Colors indicate the frequencies ω/c as labeled.

S2 Spatiotemporal coupled-mode equations

Equation of motion

The spatiotemporal dynamical equation for a system with a single mode $a(x,t)$ may be written as

$$\frac{\partial a(x,t)}{\partial t} + i\Omega a(x,t) = \int \kappa(x,x')\, s_+(x',t)\; \mathrm{d}x', \tag{5}$$

where κ is the in-coupling parameter that couples the external driving field s_+ to the internal mode. Specifically, under the locally linear dispersion of Eq. (3), this becomes

$$\frac{\partial a(x,t)}{\partial t} + i(\omega_0 - i\Gamma + v_g(\beta - \beta_0))\, a(x,t) = \int \kappa(x,x')\, s_+(x',t)\; \mathrm{d}x'. \tag{6}$$

Realizing the space-harmonic $\beta \to -i\partial_x$, this can be rewritten in the form presented in the main text:

$$\frac{\partial a(x,t)}{\partial t} + v_g \frac{\partial a(x,t)}{\partial x} + i(\omega_0 - i\Gamma - v_g\beta_0)\, a(x,t) = \int \kappa(x,x')\, s_+(x',t)\; \mathrm{d}x'. \tag{7}$$

Instead realizing the time-harmonic $\partial_t \to -i\omega$, we can write the expression for the steady-state as a purely spatial differential equation:

$$v_g \frac{\partial a(x)}{\partial x} + i(\omega_0 - \omega - i\Gamma - v_g\beta_0)\, a(x) = \int \kappa(x,x')\, s_+(x')\; \mathrm{d}x'. \tag{8}$$

We can rewrite the left-hand side as

$$\left(-i(\omega - \omega_0) + v_g \frac{\partial}{\partial x} + \Gamma - i v_g \beta_0\right) a(x) = v_g \left(\frac{\partial}{\partial x} - i\tilde{\beta}\right) a(x). \tag{9}$$

Introducing the spatial operator $\mathcal{L}$,

$$\mathcal{L}a(x) = v_g \left(\frac{\partial}{\partial x} - i\tilde{\beta}\right) a(x), \tag{10}$$

we finally obtain the compact form

$$\mathcal{L}a(x) = \int \kappa(x,x')\, s_+(x')\; \mathrm{d}x'. \tag{11}$$

Mode amplitude

To solve Eq. (11), we introduce the Green's function $G(x; x')$, defined by,

$$\mathcal{L}G(x; x') = \delta(x - x'). \tag{12}$$

For $x \neq x'$, the Green's function satisfies

$$\mathcal{L}G(x; x') = 0, \tag{13}$$

which implies,

$$\frac{\partial}{\partial x} G(x; x') = i \left(\beta_0 + \frac{\omega - \omega_0}{v_g} + i \frac{\Gamma}{v_g} \right) G(x; x') = i\tilde{\beta}\, G(x; x'). \tag{14}$$

The solution away from the source point therefore takes the form

$$G(x; x') = A\, e^{i\tilde{\beta}(x - x')}, \qquad \text{for } x \neq x'. \tag{15}$$

To determine the prefactor and the causal structure, we integrate Eq. (12) over an infinitesimal interval around x',

$$\int_{x'-\epsilon}^{x'+\epsilon} \mathcal{L}G \,\mathrm{d}x = v_g [G(x' + 0; x') - G(x' - 0; x')] = 1, \tag{16}$$

where only the derivative term contributes to the discontinuity. Restricting to a right-propagating solution enforces causality, $G(x < x') = 0$, such that $G(x' - 0; x') = 0$. This yields $G(x' + 0; x') = 1/v_g$. The Green's function can therefore be written as

$$G(x; x') = \begin{cases} \dfrac{1}{v_g}\, e^{i\tilde{\beta}(x - x')} & \text{for } x > x', \\ 0 & \text{for } x < x'. \end{cases} \tag{17}$$

Equivalently, this can be expressed in compact form using the Heaviside step function Θ,

$$G(x; x') = \frac{1}{v_g}\, e^{i\tilde{\beta}(x - x')}\, \Theta(x - x'). \tag{18}$$

The mode amplitude follows from convolution of the Green's function with the driving term,

$$a(x) = \int_{-\infty}^{+\infty} \kappa(x')\, G(x; x')\, s_+(x')\, \mathrm{d}x', \tag{19}$$

Finally, inserting Eq. (18), the mode amplitude is found as:

$$a(x) = \frac{1}{v_g} \int_0^x e^{i\tilde{\beta}(x - x')}\, \kappa(x')\, s_+(x') \mathrm{d}x'. \tag{20}$$

Input-output relation

The general form of the scattered field is written as

$$s_-(x, t) = \int \left(C(x, x')\, s_+(x', t) + d(x, x')\, a(x', t) \right) \mathrm{d}x', \tag{21}$$

where C denotes the contribution from non-resonant (background) scattering and $d(x, x')$ is a out-coupling parameter which mediates coupling from the internal mode to external radiation. In this work, we assume spatially

invariant background scattering and spatially instantaneous in- and out-coupling, such that

$$C(x,x') = C\delta(x-x'), \tag{22}$$

$$\kappa(x,x') = \kappa(x)\delta(x-x'), \tag{23}$$

$$d(x,x') = d(x)\delta(x-x'). \tag{24}$$

Under these assumptions, in the steady state, the input–output relation takes the form

$$s_-(x) = C\,s_+(x) + d(x)\,a(x). \tag{25}$$

The resonant contribution is generated by out-coupling of the guided mode back to the external channel. We write

$$s_-^{(\mathrm{res})}(x) = d(x)\,a(x). \tag{26}$$

Coupling between the external field and the guided mode is enabled by a subwavelength grating of period Λ, which provides the grating momentum $q = 2\pi/\Lambda$. In addition, coupling is restricted to a finite grating region of width W, which we represent by a window function

$$w(x) = \Theta(x)\Theta(W-x), \tag{27}$$

so that both in- and out-coupling vanish outside $x \in [0, W]$. Adopting this description, we model the in-coupling as

$$\kappa(x) = \kappa_0\,w(x)\,e^{iqx}, \tag{28}$$

and the out-coupling as

$$d(x) = d_0\,w(x)\,e^{-iqx}. \tag{29}$$

The factors $e^{\pm iqx}$ enforce grating-assisted momentum matching: the external field acquires grating momentum upon coupling into the guided mode, and the opposite phase factor appears upon re-radiation to the external channel. The overall coupling strength is constrained by energy conservation,[6,7] such that $\kappa_0 = d_0 = \sqrt{2\Gamma_{\mathrm{ext}}}$, and

$$\kappa(x) = d(x)^* = \sqrt{2\Gamma_{\mathrm{ext}}}\,e^{iqx}w(x). \tag{30}$$

Finally, by plugging Eq. (30) in Eq. (20), we obtain the general expression for the mode amplitude for the metasurface under consideration:

$$a(x) = \frac{\sqrt{2\Gamma_{\mathrm{ext}}}}{v_g}\int_0^x e^{i\tilde{\beta}(x-x')}e^{iqx'}\,w(x')\,s_+(x')\,\mathrm{d}x'. \tag{31}$$

S3 Closed-form reflection under point excitation

Input field

In general, the incident field is modeled as a diffraction-limited spot centered at position x_0, which is represented in momentum-space as

$$S_+(k_x) = S_0\,e^{-ik_x x_0}, \quad \text{for } |k_x/k_0| \le \mathrm{NA}, \tag{32}$$

where $|S_0|^2$ represents the incident power per unit k_x. Here, the in-plane wavevector is $k_x = k_0 \sin\theta$ with free-space wavevector $k_0 = \omega/c$ and incidence angle θ. The numerical aperture $\mathrm{NA} = k_{x,\max}/k_0$ sets the excitation bandwidth.

In real space, this aperture-limited momentum-space distribution produces a sinc illumination profile,

$$s_+(x') \propto \operatorname{sinc}\big(k_{x,\max}(x' - x_0)\big). \tag{33}$$

This is analogous to a diffraction-limited optical system, where an Airy disk is produced by uniform illumination of the circular entrance pupil of a microscope objective. Crucially, when the width of the resonance in k-space (governed by α) is sufficiently narrow compared to the numerical-aperture window ($\alpha/k_0 \ll \mathrm{NA}$), the incident field can be approximated as a point excitation localized at x_0,

$$s_+(x') \approx S_0\, \delta(x' - x_0), \quad \text{with } x_0 \in [0, W]. \tag{34}$$

This approximation enables the derivation of closed-form expressions for many relevant physical quantities within our framework. In the next section, we assess the accuracy of this approximation. We show that, for the system under consideration in the main text, the δ-approximation closely reproduces the numerically calculated response under sinc illumination.

Resonant scattered field

In the following, we first derive a closed-form expression for the resonant contribution $S_-^{(\mathrm{res})}(k_x)$. We start from the formal solution of the mode amplitude, Eq. (31), which, for the point-excitation approximation introduced above evaluates straightforwardly to

$$a(x) = \frac{\sqrt{2\Gamma_{\mathrm{ext}}}\, S_0}{v_g}\, e^{i\tilde{\beta}(x - x_0)} e^{iqx_0}\, \Theta(x - x_0), \tag{35}$$

with the understanding that scattering is restricted to the grating region by $w(x)$. Plugging this into Eq. (26), we obtain

$$s_-^{(\mathrm{res})}(x) = \frac{2\Gamma_{\mathrm{ext}}\, S_0}{v_g}\, e^{i(\tilde{\beta} - q)(x - x_0)} \Theta(x - x_0). \tag{36}$$

To compute the resonant scattering contribution in k-space, we integrate from x_0 to the right edge W, *i.e.*, over the modal interaction length $L = W - x_0$,

$$S_-^{(\mathrm{res})}(k_x) = \int_{x_0}^{W} s_-^{(\mathrm{res})}(x)\, e^{-ik_x x}\, \mathrm{d}x. \tag{37}$$

Substituting $u = x - x_0$ and rewriting yields

$$S_-^{(\mathrm{res})}(k_x) = \frac{2\Gamma_{\mathrm{ext}}\, S_0}{v_g} e^{-ik_x x_0} \int_0^L e^{i\tilde{\beta}u}\, e^{-i(k_x + q)u}\, \mathrm{d}u. \tag{38}$$

It is useful to define the detuning between the guided-mode wavevector and the input wave's in-plane momentum (for the $m = +1$ order) as

$$\Delta_k = (k_{\mathrm{x}} + q) - \beta = k_x - k_{x,\mathrm{res}}, \tag{39}$$

where we additionally defined the resonant in-plane wavevector in free-space $k_{x,\mathrm{res}} = \beta - q$. Using this notation, we can write

$$S_-^{(\mathrm{res})}(k_x) = \frac{2\Gamma_{\mathrm{ext}}\, S_0}{v_g}\, e^{-ik_x x_0} \int_0^L e^{-(\alpha + i\Delta_k)u} \mathrm{d}u, \tag{40}$$

which evaluates to

$$S_-^{(\mathrm{res})}(k_x) = \frac{2\Gamma_{\mathrm{ext}}\, S_0}{v_g}\, e^{-ik_x x_0}\, \frac{1 - e^{-(\alpha + i\Delta_k)L}}{\alpha + i\Delta_k}. \tag{41}$$

Reflection coefficient

The input-output relation in k-space is written as

$$S_-(k_x) = CS_+(k_x) + S_-^{(\mathrm{res})}(k_x), \tag{42}$$

and the reflection coefficient is defined as

$$r(k_x) \equiv \frac{S_-(k_x)}{S_+(k_x)}. \tag{43}$$

For the metasurfaces considered here, the off-resonant response is mirror-like, such that $C \approx -1$ over the bandwidth of interest. The reflection coefficient is then readily obtained by plugging Eqs. (32) and (41) in Eq. (43):

$$r(k_x) = -1 + \frac{2\Gamma_{\mathrm{ext}}}{v_g\,(\alpha + i\Delta_k)}\left(1 - e^{-(\alpha+i\Delta_k)L}\right). \tag{44}$$

For linear dispersion, $\Delta_\omega = v_g\Delta_k$, such that we can equivalently express the reflection coefficient in frequency-space for an ultrashort pulse as

$$r(\omega) = -1 + \frac{2\Gamma_{\mathrm{ext}}}{\Gamma + i\Delta_\omega}\left(1 - e^{-(\Gamma+i\Delta_\omega)L/v_g}\right). \tag{45}$$

In both representations, the response consists of the familiar Lorentzian dependence on the detuning as well as an exponential term which encodes the effects of a finite interaction length L. This length scale should be compared to the propagation length L_p of the quasi-guided mode. When $L \lesssim L_p$, the finite aperture produces oscillatory spectral features and effective broadening of the resonance. On the other hand, in the long-grating limit $L \gg L_p$, the finite-size factor tends to unity and the reflection coefficient reduces to the well-known one-port TCMT expression,[4]

$$r_\infty(\omega) = -1 + \frac{2\Gamma_{\mathrm{ext}}}{\Gamma + i\Delta_\omega}, \tag{46}$$

and equivalently in k-space,

$$r_\infty(k_x) = -1 + \frac{2\Gamma_{\mathrm{ext}}}{v_g(\alpha + i\Delta_k)}. \tag{47}$$

Validity of the point excitation approximation

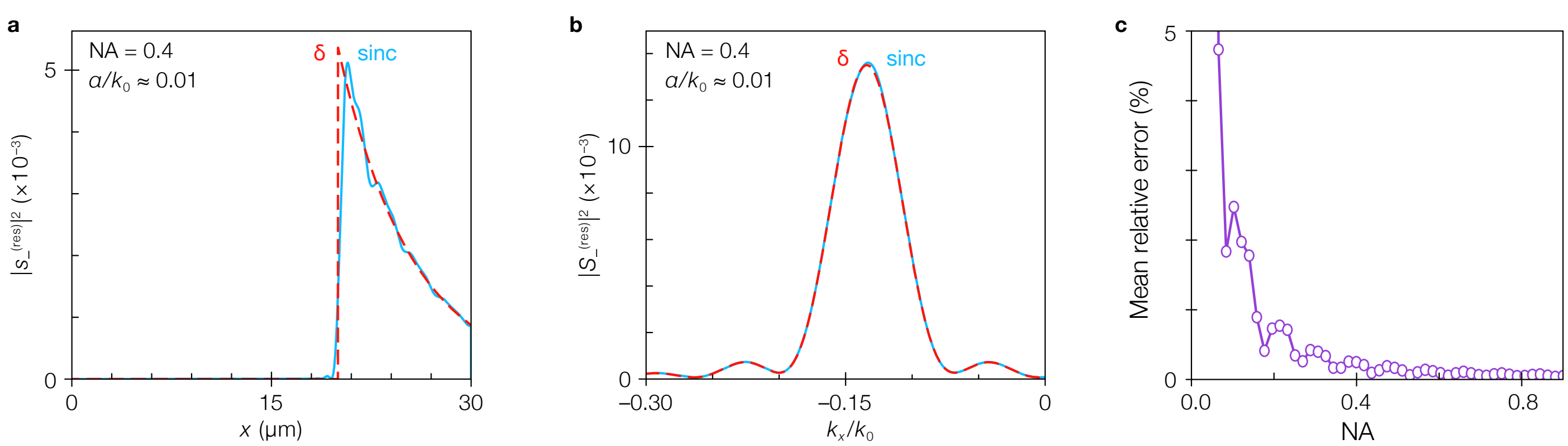

Figure S3: Evaluating the δ-function input approximation. Comparison of the NA = 0.4 sinc excitation (blue, solid) and the closed-form expressions of the infinite-NA δ-function excitation (red, dashed). **(a)** Resonant scattered field in real space, and **(b)** in momentum space evaluated at $k_0 = 9.7\ \mu\mathrm{m}^{-1}$ and $L = 10\ \mu\mathrm{m}$, where $\alpha/k_0 \approx 0.01$ **(c)** The mean relative error between $R(k_x)$ calculated using the sinc and δ-function excitations, as function of NA, shows that the error diverges as NA $\to \alpha/k_0$.

To show that the δ-approximation closely reproduces the numerically calculated response under sinc-illumination

for the system under consideration, with NA = 0.4, we compare three key parameters with and without the δ-approximation. Figures S3a,b show the resonant scattered field in real-space and in momentum-space, respectively, at $k_0 = 9.7\ \mu\mathrm{m}^{-1}$, where the interaction length is $L = 10\ \mu\mathrm{m}$ and the modal propagation length $L_p = 10.6\ \mu\mathrm{m}$, corresponding to $\alpha/k_0 \approx 0.01$ and thus satisfying $\alpha/k_0 \ll \mathrm{NA}$. While the real-space decay shows the biggest difference with a distinct oscillatory character arising from the sinc fringes, the momentum-space scattered field agrees almost perfectly. We further study the mean relative error between the reflectance ($|r(k_x)|^2$) calculated for the finite-NA sinc and infinite-NA δ-function excitations (Fig. S3c). Notably, the error exhibits a sinc-like shape, remaining below 1% when the NA is $\sim 16\times$ larger than α/k_0, while growing very large as $\mathrm{NA} \to \alpha/k_0$. Together, these results confirm the validity of the δ-approximation for the system under consideration.

S4 Finite-size effects

Resonant anti-reflection

A vanishing reflectance ($R = |r|^2 = 0$) corresponds to a destructive-interference condition in the output channel, which is modified by finite size. At resonance ($\Delta_k = 0$), the finite interaction length L introduces the factor $1 - e^{-\alpha L}$ in the resonant scattering amplitude, and the well-known critical coupling criterion $\Gamma_{\mathrm{ext}} = \Gamma_{\mathrm{int}}$ no longer corresponds to $R = 0$. Instead, the anti-reflection condition becomes

$$\frac{\Gamma_{\mathrm{ext}}}{\Gamma} = \frac{1}{2\left(1 - e^{-\alpha L}\right)}, \tag{48}$$

which generally requires stronger external coupling (more over-coupling) than the infinite-length case.

Solving Eq. (48) for L yields

$$L = -\frac{v_g}{\Gamma} \ln\!\left(1 - \frac{\Gamma}{2\Gamma_{\mathrm{ext}}}\right). \tag{49}$$

Given $\Gamma \geq 0$, a necessary condition for a real, positive solution of (49) is that $\Gamma_{\mathrm{ext}} > \Gamma/2$ (equivalently $\Gamma_{\mathrm{ext}} > \Gamma_{\mathrm{int}}$), otherwise no finite L can produce $R = 0$. It should be noted that, contrary to the infinite-length limit, the combination of parameters that yields perfect anti-reflection need not coincide with that which maximizes the stored energy U in the resonator.

Stored energy

We next derive a closed-form expression for the stored energy

$$U \equiv \int |a(x)|^2 \ \mathrm{d}x, \tag{50}$$

for $x \in [0, W]$. Using the definition of $a(x)$ (Eq. (35)), we have

$$|a(x)|^2 = 2\Gamma_{\mathrm{ext}} \left(\frac{S_0}{v_g}\right)^2 e^{-2\alpha(x - x_0)}\,\Theta(x - x_0). \tag{51}$$

Since the excitation point lies within the grating ($x_0 \in [0, W]$), the integral reduces to the interval $x \in [x_0, W]$:

$$U = 2\Gamma_{\mathrm{ext}} \left(\frac{S_0}{v_g}\right)^2 \int_{x_0}^{W} e^{-2\alpha(x - x_0)}\,\mathrm{d}x. \tag{52}$$

Substituting $u = x - x_0$ and defining $L = W - x_0$ gives

$$U = 2\Gamma_{\text{ext}} \left(\frac{S_0}{v_g}\right)^2 \int_0^L e^{-2\alpha u}\, \mathrm{d}u = 2\Gamma_{\text{ext}} \left(\frac{S_0}{v_g}\right)^2 \frac{1 - e^{-2\alpha L}}{2\alpha}. \tag{53}$$

Therefore, the stored energy is

$$U = \frac{S_0^2}{v_g} \frac{\Gamma_{\text{ext}}}{\Gamma} \left(1 - e^{-2\alpha L}\right). \tag{54}$$

In the long-grating limit $L \gg L_p$, the stored energy saturates to

$$U \to \frac{S_0^2}{v_g} \frac{\Gamma_{\text{ext}}}{\Gamma}. \tag{55}$$

Edge power flux

The finite length of the grating means that power remaining in the mode at the grating edge cannot re-radiate to free-space. We assume that the (quasi-)guided mode propagates only in the positive x-direction and that any power reaching the right edge $x = W$ leaves the patterned region, with no reflection back into a counter-propagating mode. With the normalization used here, the guided power flux at position x is

$$P(x) = v_g \left|a(x)\right|^2. \tag{56}$$

Using the expression for the mode amplitude derived above (Eq. (51)), the power crossing the right boundary is therefore

$$P_{\text{edge}} = v_g \left|a(W)\right|^2 = 2\Gamma_{\text{ext}} \frac{S_0^2}{v_g} e^{-2\alpha L}, \tag{57}$$

where $L = W - x_0$ is the distance from the excitation point to the right edge of the grating. This expression highlights the exponential suppression of edge power flux with increasing grating length.

Quality factor

In general, the quality factor of a resonance at angular frequency ω_0 is defined as

$$Q \equiv \omega_0 \frac{U}{P_{\text{loss}}} = \frac{\omega_0}{2\Gamma}, \tag{58}$$

where U is the stored energy, P_{loss} is the rate of energy dissipation. Similarly, the effective quality factor arising from the finite length of the scattering aperture is defined from the stored energy and the total power leaving this region as

$$Q_{\text{eff}}(L) = \omega_0 \frac{U}{P_{\text{loss}} + P_{\text{edge}}} = \frac{\omega_0}{2\Gamma + P_{\text{edge}}/U}. \tag{59}$$

Substituting the closed-form expressions for P_{edge} and U yields

$$Q_{\text{eff}}(L) = \frac{\omega_0}{2\Gamma} \left(1 - e^{-2\alpha L}\right) = \frac{\omega_0}{2\Gamma} \left(1 - e^{-2L/L_p}\right). \tag{60}$$

In the long-grating limit ($L \gg L_p$), the edge power flux vanishes and the quality factor approaches that of a semi-infinite grating,

$$Q_{\text{eff}} \to \frac{\omega_0}{2\Gamma}. \tag{61}$$

In the opposite limit of a size-constrained grating ($L \ll L_p$), a substantial fraction of the guided power leaves the patterned region before being re-radiated (or dissipated through absorption), reducing the effective quality factor.

In this regime, the exponential may be Taylor-expanded to yield

$$Q_{\text{eff}}(L) \simeq \frac{\omega_0}{2\Gamma}\, 2\frac{\Gamma}{v_g} L = \frac{\omega_0 L}{v_g} = \frac{\omega_0}{\Gamma}\frac{L}{L_p}. \tag{62}$$

Remarkably, in this limit, Q_{eff} is primarily governed by the mode's transit time to the edge, $t = L/v_g$. This implies that, for strongly footprint-constrained devices, high-Q requires increasing the interaction time within the fixed footprint by engineering a reduced group velocity (slow light).

Interference fringes

In addition to setting the resonance lineshape, the finite grating length also gives rise to oscillatory fringes in the k-space reflectance. Specifically, the region over which the guided mode can couple out to the far field is defined by the effective length $L = W - x_0$, and the resulting fringes arise from coherent interference between contributions emitted from different positions along the grating. This corresponds to multiplication of the real-space field by a rectangular window, whose Fourier transform produces a sinc-like modulation in k-space. As a result, the reflected intensity exhibits an interference pattern with characteristic fringe spacing

$$\Delta k_{\text{fringe}} = \frac{2\pi}{L}. \tag{63}$$

Owing to redistribution of spectral weight in k-space by this finite-aperture interference, the reflectance can locally exceed unity without implying optical gain; the scattered power remains constrained by the input power minus the dissipated power.

References

(1) Yakubovsky, D. I.; Arsenin, A. V.; Stebunov, Y. V.; Fedyanin, D. Y.; Volkov, V. S. Optical constants and structural properties of thin gold films. *Optics Express* **2017**, *25*, 25574.

(2) Liu, V.; Fan, S. S^4: A Free Electromagnetic Solver for Layered Periodic Structures. *Computer Physics Communications* **2012**, *183*, 2233–2244.

(3) Lee, S. Y.; Jeong, T. Y.; Jung, S.; Yee, K. J. Refractive Index Dispersion of Hexagonal Boron Nitride in the Visible and Near-Infrared. *Physica Status Solidi B* **2019**, *256*, 1800417.

(4) Haus, H. A., *Waves and fields in optoelectronics*; Prentice-Hall series in solid state physical electronics; Prentice-Hall: Englewood Cliffs, NJ, 1984.

(5) Haus, H.; Huang, W. Coupled-Mode Theory. *Proceedings of the IEEE* **1991**, *79*, 1505–1518.

(6) Fan, S.; Suh, W.; Joannopoulos, J. D. Temporal coupled-mode theory for the Fano resonance in optical resonators. *Journal of the Optical Society of America A* **2003**, *20*, 569.

(7) Overvig, A.; Mann, S. A.; Alù, A. Spatio-Temporal Coupled Mode Theory for Nonlocal Metasurfaces. *Light: Science & Applications* **2024**, *13*, 28.
